# Windpipe Controls *Drosophila* Intestinal Homeostasis by Regulating JAK/STAT Pathway via Promoting Receptor Endocytosis and Lysosomal Degradation

**DOI:** 10.1371/journal.pgen.1005180

**Published:** 2015-04-29

**Authors:** Wenyan Ren, Yan Zhang, Min Li, Longfei Wu, Guolun Wang, Gyeong-Hun Baeg, Jia You, Zhouhua Li, Xinhua Lin

**Affiliations:** 1 State Key Laboratory of Biomembrane and Membrane Biotechnology, Institute of Zoology, Chinese Academy of Sciences, Beijing, China; 2 University of Chinese Academy of Sciences, Beijing, China; 3 School of Optometry and Ophthalmology and Eye Hospital, Wenzhou Medical College, Wenzhou, China; 4 Department of Anatomy, National University of Singapore, Singapore; 5 Division of Developmental Biology, Cincinnati Children’s Hospital Medical Center, Cincinnati, Ohio, United States of America; 6 College of Life Sciences, Capital Normal University, Beijing, China; National Cancer Institute, United States of America

## Abstract

The adult intestinal homeostasis is tightly controlled by proper proliferation and differentiation of intestinal stem cells. The JAK/STAT (Janus Kinase/Signal Transducer and Activator of Transcription) signaling pathway is essential for the regulation of adult stem cell activities and maintenance of intestinal homeostasis. Currently, it remains largely unknown how JAK/STAT signaling activities are regulated in these processes. Here we have identified *windpipe* (*wdp*) as a novel component of the JAK/STAT pathway. We demonstrate that Wdp is positively regulated by JAK/STAT signaling in *Drosophila* adult intestines. Loss of *wdp* activity results in the disruption of midgut homeostasis under normal and regenerative conditions. Conversely, ectopic expression of Wdp inhibits JAK/STAT signaling activity. Importantly, we show that Wdp interacts with the receptor Domeless (Dome), and promotes its internalization for subsequent lysosomal degradation. Together, these data led us to propose that Wdp acts as a novel negative feedback regulator of the JAK/STAT pathway in regulating intestinal homeostasis.

## Author Summary

Effective tissue homeostasis requires a proper balance between the removal of dead cells and production of new cells. Due to environmental challenges, the *Drosophila* midgut epithelial cells are damaged from time to time and intestinal stem cells (ISC) can accelerate their proliferative rate to replace the lost midgut epithelium. The JAK/STAT pathway plays essential roles in these progresses. Upon damage, Upd ligands produced by dying enterocytes (ECs) activate JAK/STAT signaling in ISCs to promote their proliferation and differentiation. However, after damage how JAK/STAT signaling is switched from a highly active state to a homeostatic state is not yet fully understood. In this study, we identified the leucine rich repeats (LRR) protein Windpipe (Wdp) as a novel negative feedback regulator of JAK/STAT signaling during intestinal development. Wdp expression was induced by high levels of JAK/STAT signaling in intestines. And loss of Wdp leads to midgut homeostasis loss and increased ISC proliferation. Furthermore, we found Wdp in turn negatively regulates JAK/STAT signaling activity through promoting Domeless receptor endocytosis and lysosomal degradation. In this way, high levels of JAK/STAT signaling is switched off by Wdp, which ensure ISCs return to the homeostatic state after tissue damage.

## Introduction

The JAK/STAT pathway is evolutionarily conserved from *Drosophila* to mammals, and plays important roles in various developmental processes including cellular proliferation, innate immune response and stem cell development [1–4]. Dysregulation of the JAK/STAT pathway is associated with many human diseases, such as immune disorders and cancers [5–7]. Therefore, the JAK/STAT pathway is tightly controlled by various regulators and mechanisms to ensure proper signaling. While the core components of this pathway are well-documented, it is less understood how the duration of its signal activity is temporally regulated.

*Drosophila* is an excellent model to investigate the regulation of JAK/STAT signaling. Compared with various isoforms of the JAK/STAT pathway components in mammals [8–10], *Drosophila* has a relatively simple signal transduction cascade: a one-pass transmembrane receptor, Domeless (Dome) [11, 12]; a tyrosine JAK kinase, Hopscotch (Hop) [13]; a transcription factor, STAT92E [14, 15]; and three different ligands including Unpaired (Upd) [16], Upd2 [17], and Upd3 [18]. In the canonical pathway, binding of Dome receptor with its extracellular ligands induces Dome dimerization or oligomerization, which leads to juxtaposition of Hop. Hop molecules cross-phosphorylate each other and then phosphorylate Dome to generate docking sites for cytoplasmic STAT92E. Once bound to the Dome/Hop complex, STAT92E molecules are phosphorylated, form dimers, and then translocate into the nucleus, where they bind to defined STAT92E binding sites, and regulate the transcription of downstream target genes [1, 19]. This signaling transduction is under tight control at multiple steps to avoid improper signal activation [1, 9]. Several negative feedback regulators such as Socs36E and Ptp61F are identified to be involved in switching off JAK/STAT signaling during developmental processes [20–22].

JAK/STAT signaling pathway plays important roles in *Drosophila* adult midgut homeostasis and tissue regeneration. Due to dietary stress, tissue injury, or pathogen infection, intestinal epithelial cells turn over rapidly and midgut homeostasis is maintained by intestinal stem cells (ISC). A basally localized ISC divides asymmetrically to give rise to a renewed ISC and a non-dividing, undifferentiated enteroblast (EB). EBs then differentiate into either absorptive enterocytes (EC) or secretory enteroendocrine cells (ee) [23, 24]. Several signaling pathways including Notch, JAK/STAT, EGFR, Hippo, insulin, BMP and Wnt have been shown to regulate the maintenance, proliferation, and differentiation of ISCs [23–41]. Under physiological conditions, JAK/STAT signaling promotes ISC proliferation and is also required for the differentiation of ECs and ee cells [26–28, 42, 43]. In addition, the JAK/STAT pathway plays crucial roles during midgut regeneration. After bacterial infection or physical injury, the expression of Upd ligands such as Upd3 is induced. Secreted Upd ligands from ECs activate JAK/STAT signaling in ISCs, which rapidly increase their proliferation rate to replenish the damaged midgut epithelium [26, 44–46]. However, the mechanisms of how highly activated JAK/STAT signaling returns to normal levels after injury remain poorly understood.

*Drosophila* Windpipe (Wdp) is a single-pass transmembrane protein containing four leucine-rich repeats (LRR) in the extracellular domain, and is highly expressed in the developing trachea [47]. Currently, the biological function of this protein has not been defined. In this study, we have identified *wdp* as a novel component of the JAK/STAT pathway. We showed that Wdp is positively regulated by JAK/STAT signaling. Loss of *wdp* results in disruption of midgut homeostasis under physiological conditions, and potentiates tissue regeneration under damage conditions. Conversely, ectopically expressed Wdp negatively regulates JAK/STAT signaling. Importantly, we demonstrate that Wdp can promote Dome internalization and subsequent lysosomal degradation. Together, we propose that Wdp controls intestinal homeostasis by interfering with JAK/STAT signaling activity via a negative feedback mechanism.

## Results

### Wdp expression is positively regulated by JAK/STAT signaling in *Drosophila* midguts

Although the JAK/STAT pathway is required for regulating midgut homeostasis under physiological conditions and damage-induced tissue regeneration, how JAK/STAT signaling executes its functions during these processes remains largely unknown. So far, only a few STAT92E target genes including *Socs36E*, *zfh1*, and *chinmo*, have been identified [48–50]. To further explore the regulatory mechanism of the JAK/STAT pathway in midguts, we performed ChIP-Seq experiments aimed at identifying novel downstream targets of the JAK/STAT pathway. ChIP-Seq experiments were carried out in intestines ectopically expressing Upd and STAT92E in the progenitor cells (*esg* ^*ts*^ *>upd*; *STAT)*. In these experiments, we identified about 200 candidates with at least one peak (p<0.01) in the gene regulatory region. The previously well-characterized JAK/STAT downstream targets, including *Domeless* [51], *Socs36E* [20], and *STAT92E* [52, 53], were recovered in our experiments (S1 Table), indicating that our ChIP approach is workable to identify potential novel targets.

**Fig 1:**
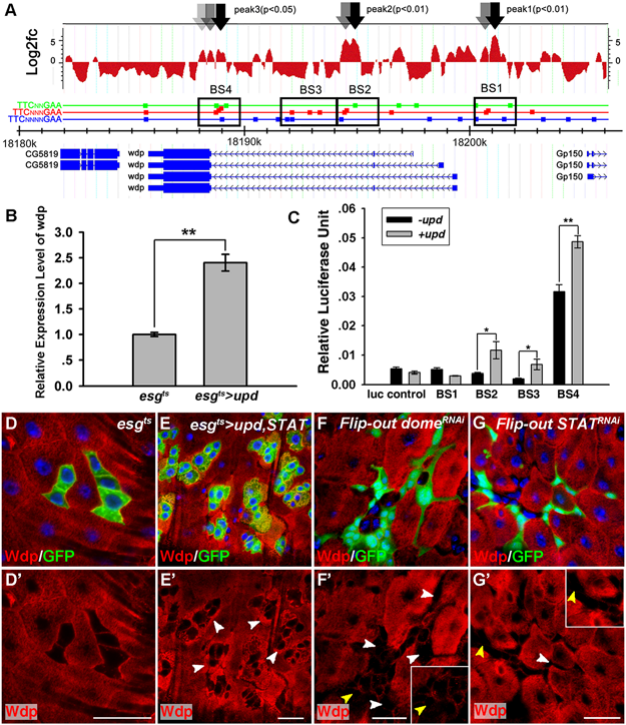
Wdp expression is positively regulated by JAK/STAT signaling in *Drosophila* intestines. (A) ChIP analysis was performed to monitor the binding of STAT92E to *wdp* genomic regions with STAT92E antibody using adult intestines expressing Upd and STAT92E under the *esg^ts^* driver for 10 days at 29°C. The localization of four putative STAT92E-binding sites (BS1-4) is indicated by a black square frame. The square boxes with green, red or blue colors represent putative STAT92E binding sites localized in *wdp* genomic region with 2, 3 or 4 spacers respectively. (B) *wdp* mRNA expression was obviously increased in *esg^ts^>upd* intestines at 29°C for 7 days using RT-qPCR quantification. Mean ± SD are shown. **p<0.01. (C) The relative activity of the indicated luciferase vectors, which contain different putative STAT92E binding sites (BS1-4) from *wdp* genomic regions, upon addition of Upd expressing cells. Mean ± SD are shown. *p<0.1, **p<0.01. (D and D’) Wdp (red, by Wdp) is ubiquitously expressed in both small progenitor cells and large nuclei ECs in control midguts at 29°C for 7 days. (E and E’) Wdp expression (red, by Wdp) was significantly increased around the GFP+ clusters (arrowheads) in *esg^ts^* >*upd*, *STAT* midguts at 29°C for 7 days. (F and F’) Wdp expression (red, by Wdp) was reduced in the Flip-out clones (arrowheads) knocking down Dome at 29°C for 7 days. Square box is the enlarged image of the position labeled by yellow arrowhead. (G and G’) Wdp expression (red, by Wdp) was reduced in the Flip-out clones (arrowheads) knocking down STAT at 29°C for 7 days. Square box is the enlarged image of the position labeled by yellow arrowhead. Blue indicates DAPI staining. Scale bars, 20μm.

From the candidate genes, *wdp*, a gene previously shown to be highly expressed in the developing trachea [47], was identified. Wdp was ranked in the top 10% through our bioinformatics analysis of ChIP results. At least 3 significant peaks (p<0.01) containing conserved STAT92E binding sites (TTCN3/4GAA) [15] were found in the 5’ UTR and genomic region of *wdp* (Fig 1A). Moreover, *wdp* mRNA levels as determined by RT-qPCR were increased in response to ectopic JAK/STAT signaling in *esg* ^*ts*^>*upd* intestines (Fig 1B). To further analyze the transcriptional regulation of *wdp* by JAK/STAT signaling, we identified four potential STAT92E binding sites (BS1-, BS2-, BS3- and BS4), and generated luciferase reporters that contain these potential binding sites. With the addition of Upd expressing S2 cells, the luciferase activity in cells transfected with BS2-, BS3- or BS4- luciferase constructs was obviously increased (Fig 1C). These results suggest that the expression of Wdp might be regulated by JAK/STAT signaling through BS2, BS3 and BS4 binding sites.

Next we wanted to determine whether the expression of Wdp is regulated by JAK/STAT signaling *in vivo* in *Drosophila* posterior midgut. First, the expression pattern of Wdp was examined by immunostaining with anti-Wdp antibody. The specificity of anti-Wdp antibody was verified (S1G–S1I Fig). Then we determined Wdp expression pattern in midguts and imaginal discs (S1 Fig). Wdp was ubiquitously expressed in wild-type intestines (Fig 1D, 1D’ and S1A–S1D’ Fig). When we used *esg* ^*ts*^ to overexpress Upd and STAT in progenitor cells, we found Wdp protein levels were increased around the GFP^+^ clusters (Fig 1E and 1E’). Furthermore, we blocked JAK/STAT signaling activity by expressing *dome RNAi* or *STAT RNAi*, and found Wdp expression was reduced in *dome RNAi*/*STAT RNAi* expressing cells (Fig 1F–1G’ and S2A–S2D’ Fig). Besides, Wdp expression was also reduced in *STAT92E* ^*06346*^ mutant clones (S2E–S2F’ Fig). To further confirm the regulation of Wdp by JAK/STAT signaling in ISCs, we first generated *Notch* ^*264-39*^ mutant ISC clusters and found Wdp was mainly localized on the cell membrane in ISCs. However, Wdp expression levels were reduced in the ISC clusters deficient for stat92E with simultaneous Notch knockdown (S2G–S2H’ Fig), indicating that Wdp expression was reduced in JAK/STAT signaling deficient ISCs. Taken together, these data indicate that *wdp*, as a putative target of STAT92E, is positively regulated by JAK/STAT signaling in *Drosophila* intestines.

### Loss of *wdp* disrupts midgut homeostasis under physiological conditions and potentiates tissue regeneration under damage conditions

Next, we examined the possible functions of *wdp* in midgut homeostasis. We generated 2 alleles of *wdp* mutants by imprecise excision of *P{wHy}wdp* ^*DG23704*^ (S1E Fig) and selected *wdp* ^*1*^ for further experiment. *wdp* ^*1*^ is likely a functional null mutant, as half of the *wdp* coding sequence is removed. Consistently, *wdp* transcription in *wdp* ^*1/1*^ homozygotes was abolished compared with *WT* (S1F Fig). Homozygous *wdp* ^*1/1*^ flies are semi-lethal with a few escapers displaying no visual phenotypes.

**Fig 2:**
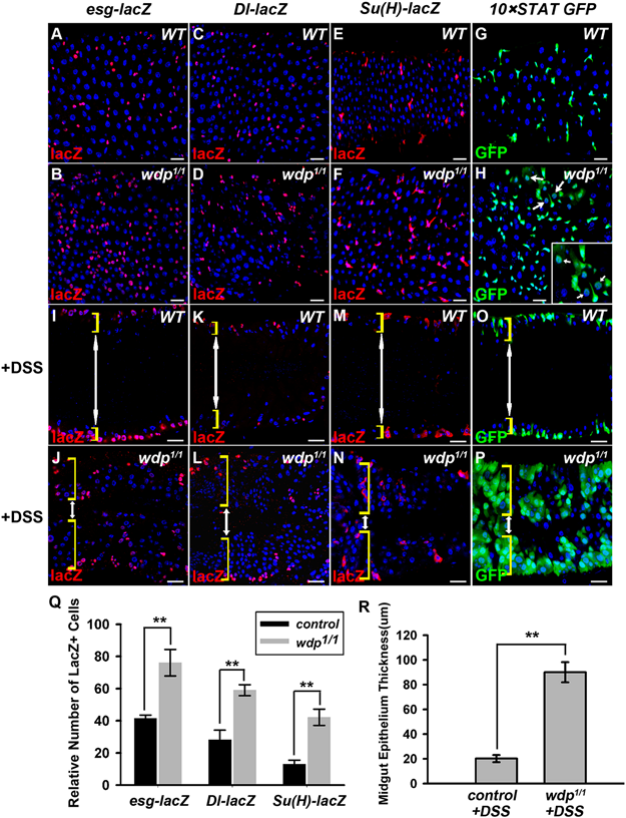
Loss of *wdp* disrupts midgut homeostasis under normal conditions and potentiates DSS-induced midgut regeneration. (A and B) The progenitor cells (red, by *esg-lacZ*) in control (A) or *wdp^1/1^* adult midguts (B) at 25°C for 7 days. (C and D) ISCs (red, by *Dl-lacZ*) in control (C) or *wdp^1/1^* adult midguts (D) at 25°C for 7 days. (E and F) EBs [red, by *Su(H)GBE-lacZ*] in control (E) or *wdp^1/1^* adult midguts (F) at 25°C for 7 days. (G and H) *10×STAT GFP* positive cells in control (G) or *wdp^1/1^* adult midguts (H) at 25°C for 7 days. The appearance of 10×STAT GFP in putative ECs was observed in *wdp^1/1^* mutants (arrows in H). Square box in H shows the enlarged image of the position labeled by white arrows. (I and J) The progenitor cells (red, by *esg-lacZ*) in the cross-section of midgut epithelium from control (I) or *wdp^1/1^* adults (J) upon 3% DSS treatment at 29°C for 4 days. Both sides of the midgut epithelium are shown. The yellow brackets indicate the intestinal wall and the white double-headed arrows indicate the intestinal lumen. (K and L) ISCs (red, by *Dl-lacZ*) in the cross-section of midgut epithelium from control (K) or *wdp^1/1^* adults (L) upon 3% DSS treatment at 29°C for 4 days. (M and N) EBs [red, by *Su(H)GBE-lacZ*] in the cross-section of midgut epithelium from control (M) or *wdp^1/1^* adults (N) upon 3% DSS treatment at 29°C for 4 days. (O and P) *10×STAT GFP* positive cells in the cross-section of midgut epithelium from control (O) or *wdp^1/1^* adults (P) upon 3% DSS treatment at 29°C for 4 days. (Q) Quantification of the relative number of *esg-lacZ*, *Dl-lacZ* or *Su (H)-lacZ* positive cells in *wdp^1/1^* adult midguts at 25°C for 7 days. Mean±SD are shown. n = 5–10 intestines. **p<0.01. (R) The thickness of midgut epithelium (μm) from control or *wdp^1/1^* adults upon 3% DSS treatment at 29°C for 4 days. Mean±SD are shown. n = 8–10 intestines. **p<0.01. Blue indicates DAPI staining. Scale bars, 20μm.

To examine the function of Wdp in the posterior midgut, we used *esg-lacZ*, *Dl-lacZ* and *Su(H)GBE-lacZ* to mark progenitor cells, ISCs and EBs respectively. Compared with the controls, the number of *esg-lacZ* positive cells was significantly increased in *wdp* ^*1/1*^ mutant intestines (Fig 2A, 2B and 2Q). Similar phenotype in *wdp* ^*1/2*^ trans-heterozygotes was observed (S3E–S3G Fig), excluding the existence of possible background mutations. We also found an increased number of *Dl-lacZ* and *Su(H)GBE-lacZ* positive cells in *wdp* ^*1/1*^ intestines (Fig 2C–2F and 2Q). Moreover, the number of *10×STAT GFP* positive cells was obviously increased (Fig 2G and 2H). In addition, 10×STAT GFP seems to appear in the large putative EC cells (arrows in Fig 2H). These results suggest that midgut homeostasis is disrupted upon *wdp* loss under normal conditions.

We further examined the roles of Wdp under damage conditions. Midgut regeneration of *wdp* ^*1/1*^ adults was monitored in response to dextran sulfate sodium (DSS) feeding, which is used to investigate ISC proliferation and tissue regeneration upon damage [54]. Adult flies aged at 3 or 4 days were treated with 3% DSS for 4 days. Under DSS treatment, *wdp* ^*1/1*^ adults showed dramatic hyperplasia and extensive multilayering of the midgut epithelium compared with controls (Fig 2I–2P and 2R). Moreover, the number of progenitor cells and *10×STAT GFP* positive cells was also increased (Fig 2I–2P), indicating that tissue damage induced midgut regeneration was abnormally enhanced in the absence of *wdp*. Collectively, these data indicate that loss of *wdp* disrupts midgut homeostasis under normal conditions and potentiates tissue regeneration under damage conditions.

### Wdp inhibits ISC proliferation and restricts ISC overproliferation induced by ectopic JAK/STAT signaling

**Fig 3:**
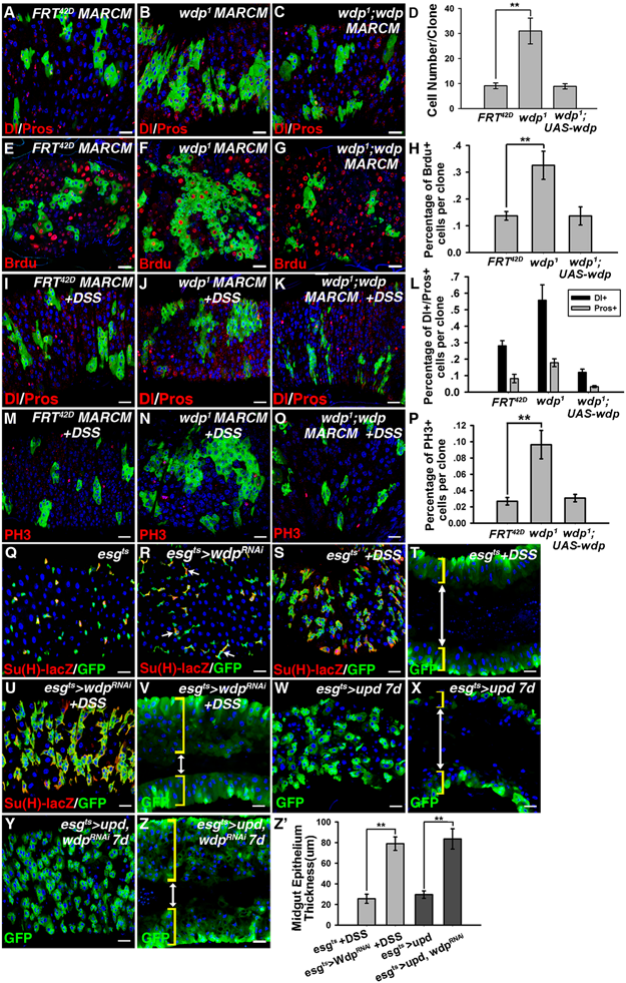
Wdp inhibits ISC proliferation and restricts the ISC overproliferation caused by ectopic JAK/STAT signaling. (A-C) Dl/Pros in MARCM control clones (A), MARCM clones of *wdp^1^* cells (B) and MARCM clones of *wdp^1^* cells with simultaneous Wdp expression (C). The overproliferation of ISC observed in *wdp^1^* mutant MARCM clones (B) was rescued in the presence of transgenic *wdp* (C). (D) Quantification of clone size 6D ACI, including control, *wdp^1^* mutant and *wdp^1^* mutant while expressing transgenic *wdp* MARCM clones. Mean±SD are shown. n = 9 intestines. **p<0.01. (E-G) Brdu incorporation (Red, by Brdu) in MARCM control clones (E), *wdp^1^* mutant clones (F) and *wdp^1^* mutant clones with simultaneous Wdp expression (G). The increased Brdu incorporation in *wdp^1^* mutant MARCM clones (F) was rescued with the simultaneous Wdp expression (G). (H) Quantification about the percentage of Brdu incorporation per clone with different genotypes. Mean±SD are shown. n = 6–9 intestines. **p<0.01. (I-P) Adult flies with control (I and M), *wdp^1^* mutant (J and N) and *wdp^1^* mutant while expressing transgenic Wdp (K and O) MARCM clones were treated with DSS at 29°C for 4 days. Under stress conditions, the number of Dl/Pros positive cells within *wdp^1^* clones was also increased compared with controls (I and J). The overabundance of PH3 positive cells due to *wdp* depletion (M and N) was suppressed in the presence of transgenic *wdp* (O). (L) Quantification about the percentage of Dl+ and Pros+ cells per clone with indicated genotypes under DSS treatment. Mean±SD are shown. n = 8–10 intestines. (P) Quantification about the percentage of PH3+ cells per clone in intestines containing different MARCM clones under DSS treatment. Mean±SD are shown. n = 11–15 intestines. **p<0.01. (Q and R) The number of GFP+ cells was mildly increased upon knockdown of *wdp* using *Su(H)GBE-lacZ; esg^ts^* driver (R) compared with controls (Q) at 29°C for 7 days. (S-V) Adult flies of *esg^ts^* or *esg^ts^* >*wdp RNAi* were treated with 3% DSS for 4 days at 29°C. Cross-section of midgut epithelium with the indicated genotypes was shown in T and V. Upon DSS treatment, the number of GFP+ clusters (S and U) as well as the thickness of midgut epithelium (T and V) from *esgts >wdp RNAi* midguts were significantly increased compared with controls. The intestinal lumen is indicated by white double-headed arrows and the intestinal wall by yellow brackets. (W-Z) The overproliferation of ISCs caused by *upd* expression (W and X) using the *esg^ts^* driver was strikingly enhanced with simultaneous *wdp* knockdown (Y and Z). Cross-section of midgut epithelium with the indicated genotypes was shown in X and Z. (Z’) Quantification of midgut epithelium thickness (μm) with the indicated genotypes. Mean±SD are shown. n = 7–10 intestines. **p<0.01. Blue indicates DAPI staining. Scale bars, 20μm.

We further examined whether Wdp is involved in regulating ISC activity. First, mosaic analysis with repressible cell marker (MARCM) approach was used to generate GFP positively marked clones for *wdp* ^*1*^ mutants [55]. The control ISC clones contained an average of 7–8 cells per clone 6 days after clone induction (ACI) (Fig 3A, 3D and 3E). In contrast, the *wdp* ^*1*^ mutant ISC clones contained up to 30 cells per clone 6d ACI (Fig 3B, 3D and 3F). Moreover, the number of Dl/Pros positive cells, which mark ISC/ee respectively, was increased in *wdp* ^*1*^ mutant ISC clones compared with controls (Fig 3A and 3B). In addition, Brdu incorporation within *wdp* ^*1*^ mutant clones was also enhanced (Fig 3E, 3F and 3H), suggesting that loss of Wdp led to the increased proliferation of ISCs.

We also examined the role of *wdp* in regulating ISC proliferation under damage conditions. Adult flies carrying MARCM clones of various genotypes were fed with DSS. Consistently, we found the size of *wdp* ^*1*^ mutant clones was obviously enlarged, and the number of Dl/Pros positive cells was also increased within *wdp* ^*1*^ clones compared with controls under damage conditions (Fig 3I, 3J and 3L). Similarly, the number of PH3 positive cells per gut was enhanced in the intestines containing *wdp* ^*1*^ mutant clones (Fig 3M, 3N and 3P). It is important to mention that the increased ISC proliferation of *wdp* ^*1*^ mutants under physiological conditions or damage conditions could be rescued by simultaneous *wdp* expression (Fig 3C, 3G, 3K and 3O), confirming that the observed defects were derived from loss of Wdp activity.

We also knocked down Wdp in progenitor cells by expressing *wdp RNAi* driven by *esg* ^*ts*^. A mild increase in GFP^+^ cells was observed when *wdp* was knocked down in the progenitor cells (Fig 3Q and 3R). Moreover, when the *wdp* knockdown flies were treated with DSS, the number of *GFP+* cells was increased, and the midgut epithelium exhibited extensive multilayering compared with controls (Fig 3S–3V). This suggests that the tissue damage induced ISC proliferation was enhanced in the absence of *wdp*. Taken together, the above data derived from *wdp* mutant clones and RNAi experiments indicate that *wdp* restricts ISC proliferation under normal and regenerative conditions.

As *wdp* expression could be induced by ectopic JAK/STAT signaling in the intestines, we examined its role under high levels of JAK/STAT signaling. When *upd* was ectopically expressed using the *esg* ^*ts*^ driver, ISC proliferation was obviously increased (Fig 3W and 3X). Surprisingly, simultaneous knockdown of *wdp* enhanced the excessive ISC proliferation induced by ectopic Upd expression, as determined by the increase in GFP^+^ cells and a thickened midgut epithelium (Fig 3Y–3Z’). These results indicate that Wdp restricts ISCs from excessive proliferation caused by ectopic JAK/STAT signaling.

### Wdp downregulates JAK/STAT signaling activity

**Fig 4:**
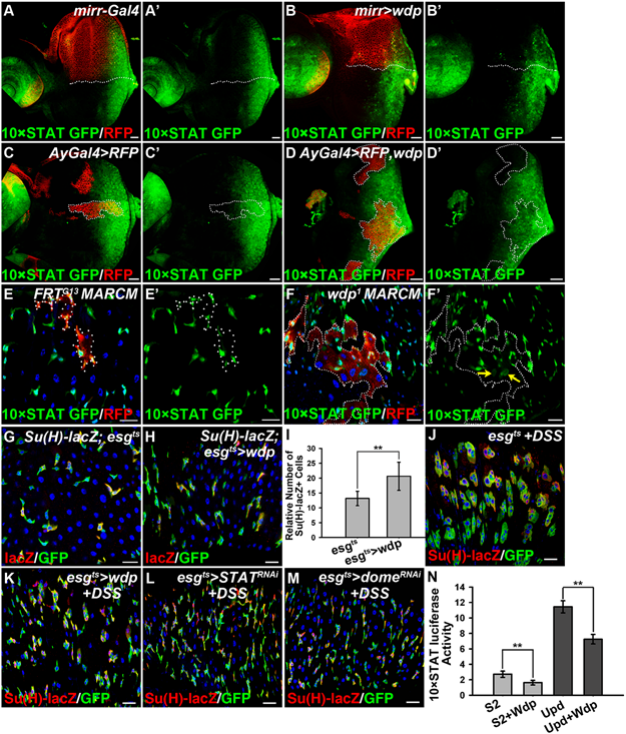
Wdp negatively regulates JAK/STAT signaling. (A and A’) In WT, 10×STAT GFP is highly expressed throughout the posterior part of early 3rd instar eye discs. All the eye discs shown here are oriented anterior left, dorsal up. D/V boundary is shown by the dotted line. (B and B’) The levels of 10×STAT GFP (B’) were reduced in the dorsal compartment of early 3^rd^ instar larva eye discs upon *wdp* expression using *mirrorGal4*. CD8-mRFP was used to mark the dorsal compartment. (C-C’) The activity of 10×STAT GFP in early 3^rd^ instar larva eye discs bearing the control flip-out clones (*Act>y+>Gal4*, *UAS-RFP*) marked by the presence of RFP and dotted lines. (D-D’) The activity of 10×STAT GFP was decreased in Wdp overexpressing clones marked by RFP expression and dotted lines compared with surrounding WT cells in early 3^rd^ instar larva eye discs. (E-F’) 10×STAT GFP was used to monitor the activity of JAK/STAT signaling in intestinal MARCM clones marked by the presence of RFP. The activity of 10×STAT GFP was increased in *wdp* ^1^ MARCM clones compared with surrounding wild-type cells (F and F’). Yellow arrows in F’ indicate the appearance of 10×STAT GFP in putative ECs. Dotted lines denote the position of MARCM clone cells. (G-I) A slightly increased number of EBs was found upon overexpression of *wdp* (H) using *esg^ts^* driver at 29°C for 10 days compared with controls (G). I shows quantification of *Su(H)GBE-lacZ* positive cells. Mean±SD are shown. n = 8 intestines. **p<0.01. (J-M) Adult flies of *esg^ts^* (J), *esgts>wdp* (K), *esgts>STAT RNAi* (L) or *esgts>Dome RNAi* (M) were treated with DSS for 4 days at 29°C before dissection. Overexpression of *wdp* using the *esg^ts^* driver (K) blocked the formation of large GFP+ clusters caused by DSS treatment. The phenotype was reminiscent of flies with STAT or Dome knockdown using *esg^ts^* driver (L and M) when fed with DSS. (N) The activity of *10×STAT luciferase* reporter in S2 cells transfected with *UAS-wdp*. Wdp expression was able to suppress the basal *luciferase* activity as well as Upd-induced upregulation of *10×STAT luciferase* activity. Mean±SD are shown. **p<0.01. Blue indicates DAPI staining in E-M. Scale bars, 20μm.

To gain insights into the mechanistic role of *wdp* in the JAK/STAT pathway, we examined its potential regulation of JAK/STAT signaling in other developmental processes. Eye imaginal disc is a good model to investigate JAK/STAT signaling [56, 57]. We detected the expression of Wdp in 3^rd^ instar eye discs (S1J Fig). 10×STAT GFP is used as the signaling readout [58], which is detected throughout the posterior part of early 3rd instar eye discs (Fig 4A and 4A’). When *wdp* was ectopically expressed using *mirror-Gal4* in the dorsal compartment, the levels of 10×STAT GFP were obviously reduced (Fig 4B and 4B’). Consistently, the activity of 10×STAT GFP was also decreased in the flip-out clones overexpressing Wdp compared with surrounding WT cells (Fig 4C–4D’). On the contrary, we detected enhanced expression region of 10×STAT GFP in the *wdp* ^*1/1*^ homozygous eye discs (S4A–S4C Fig). Moreover, Wdp knockdown using *mirror-gal4* also led to the enlarged 10×STAT GFP region in the dorsal compartment of 3^rd^ instar eye discs (S4D–S4D‴ Fig). These data indicate that Wdp negatively regulates JAK/STAT signaling in eye discs. In contrast, Wingless (Wg), Hedgehog (Hh), and Decapentaplegic (Dpp) signaling pathways were not affected when *wdp* was ectopically expressed (S5 Fig), suggesting that Wdp mainly regulates JAK/STAT signaling in imaginal discs.

As mentioned above, loss of Wdp could potentiate Upd induced ISC proliferation (Fig 3W–3Z’), implying its regulation of JAK/STAT signaling in posterior midguts. To verify this hypothesis, we examined JAK/STAT signaling by detecting the activity of 10×STAT GFP in RFP positively marked intestinal MARCM clones. In *wdp* ^*1*^ mutant clones, the levels of 10×STAT GFP were mildly increased when compared with surrounding wild-type cells (Fig 4F and 4F’). Moreover, in *wdp* ^*1*^ mutant clones we detected 10×STAT GFP in the large cells (putative EC cells) as well as in small progenitors (arrows in Fig 4F’), implying JAK/STAT signaling maybe abnormally activated in ECs. However, in control clones there seems no obvious difference of 10×STAT GFP activity between RFP+ clones and RFP- cells. Besides, 10×STAT GFP was restricted in small progenitors (Fig 4E and 4E’). Furthermore, we examined the destabilized 10×STAT DGFP reporter [58] in *wdp* ^*1/1*^ intestines and found the activity of 10×STAT DGFP was obviously increased compared with controls (S4E–S4F’ Fig). Consistently, 10×STAT DGFP also appeared in large ECs in *wdp* ^*1/1*^ homozygotes (S4E–S4F’ Fig). These results suggest that JAK/STAT signaling was upregulated in the absence of *wdp*. Meanwhile, we used the *esg* ^*ts*^ driver to overexpress *wdp* in the progenitor cells and found mild increase of EBs under normal conditions (Fig 4G–4I). However, when *esg* ^*ts*^ *>wdp* flies were treated with DSS, the damage-induced tissue regeneration was suppressed. In contrast to the large GFP+ clusters containing both small diploid progenitors and large polyploid cells in the controls, there were no large GFP+ cells observed in the clusters of the *esg* ^*ts*^ *>wdp* midguts (Fig 4J and 4K). This was reminiscent of intestines with deficient JAK/STAT signaling by STAT or Dome knockdown under DSS treatment (Fig 4L and 4M). Altogether, these data suggest that Wdp could interfere with JAK/STAT signaling in posterior midguts.

We further assessed the activity of *10×STAT luciferase* reporter in S2 cells transfected with *wdp*. Consistently, we found Wdp expression was able to suppress the basal *10×STAT luciferase* as well as Upd-induced upregulation of *10×STAT luciferase* activity (Fig 4N). Taken together, the above data derived from different tissues indicate that Wdp is a negative regulator of the JAK/STAT signaling pathway.

### Wdp acts downstream of Upd but upstream of Hop

**Fig 5:**
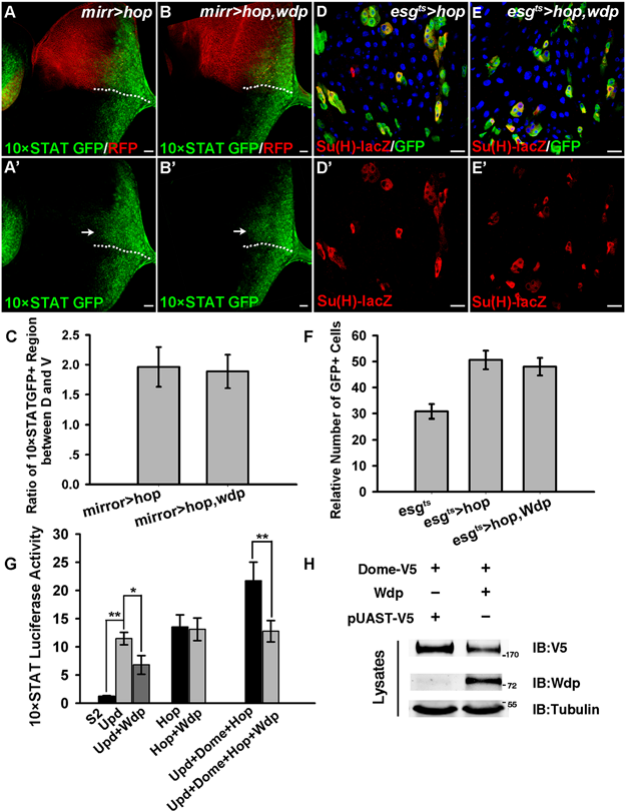
Wdp functions downstream of Upd but upstream of Hop. (A and A’) The activity and the expression regions of 10×STAT GFP were noticeably enhanced (arrow in A’) when *hop3w* was ectopically expressed using *mirrorGal4* in the dorsal compartment marked by CD8-mRFP of early 3^rd^ instar larva eye discs. D/V boundary is shown by the dotted line. All the eye discs shown here are oriented dorsal up, posterior right. (B and B’) Simultaneous expression of *wdp* was unable to suppress the increased 10×STAT GFP activity due to the *hop3w* overexpression in the dorsal compartment (arrow in B’). (C) Quantification about the ratio of 10×STAT GFP expression region (along the A-P axis) between dorsal and ventral part in early 3^rd^ instar eye discs with indicated genotypes. Mean±SD are shown. n = 9–10 discs. (D-D’) Ectopic expression of *hop3w* using the *esg^ts^* driver promotes ISC proliferation at 29°C for 7 days. (E-E’) Simultaneous expression of *wdp* cannot suppress overproliferation of ISC caused by ectopic *hop3w* expression using the *esg^ts^* driver at 29°C for 7 days. (F) Quantification of the relative number of *esgts>GFP* cells with indicated genotypes. Mean±SD are shown. n = 12–15 intestines. (G) The increased activity of *10×STAT luciferase* due to Upd expression can be suppressed by cotransfection of UAS-*wdp*. However, *wdp* overexpression can’t block the constitutive activation of JAK/STAT signaling caused by *hop*. The increased *10×STAT luciferase* activity resulting from cotransfection of UAS-*upd*, *dome* and *hop* was obviously suppressed in the presence of *wdp*. Mean±SD are shown. *p<0.1, **p<0.01. (H) S2 cells transfected with UAS-*dome-V5* alone or along with *wdp* were lysed and assessed for total Dome levels using V5 antibody. Blue indicates DAPI staining in D and E. Scale bars, 20μm.

To determine the level at which Wdp modulates JAK/STAT signaling, we examined the epistatic relationship between Wdp and the JAK/STAT pathway components. When *hop* was ectopically expressed using *mirrorGal4* in eye discs, we observed increased JAK/STAT activity, as determined by an expanded expression region of 10×STAT GFP in the dorsal compartment which is marked by CD8-mRFP (Fig 5A and 5A’, arrow). Simultaneous expression of *wdp* failed to suppress the elevated JAK/STAT signaling caused by *hop* overexpression (Fig 5B, 5B’ and 5C), suggesting that Wdp acts upstream of Hop. Consistent with previous study [28], ectopic expression of Hop in midguts using the *esg* ^*ts*^ driver led to weak expansion of *esg* positive cells (Fig 5D and 5D’). ISC over-proliferation caused by *hop* expression was not affected in the presence of *wdp* (Fig 5E, 5E’ and 5F). Thus, these epistatic experiments performed in both the midguts and eye discs placed Wdp upstream of Hop.

To further confirm the genetic epistasis between Wdp and Hop, we assessed the activity of *10×STAT luciferase* reporter in S2 cells cotransfected with *wdp* and *hop-V5* vectors. Consistently, the increased *10×STAT luciferase* activity resulting from *hop* expression could not be blocked by cotransfection *of wdp*. However, ectopic expression of *wdp* was able to suppress the enhanced activity of *10×STAT luciferase* caused by Upd expression, indicating that Wdp acts downstream of Upd. In addition, increased *10×STAT luciferase* activity resulting from simultaneous transfection of UAS-*upd*, *dome*, and *hop* was significantly suppressed by cotransfection with *wdp* (Fig 5G). Moreover, we found Wdp expression could autonomously suppress the upregulation of JAK/STAT signaling caused by ectopic Upd expression in imaginal discs (S6 Fig), further confirming that Wdp functions downstream of Upd. Taken together, Wdp acts upstream of Hop but downstream of Upd to inhibit JAK/STAT signaling.

### Wdp interacts with Domeless and promotes its endocytosis and lysosomal degradation

**Fig 6:**
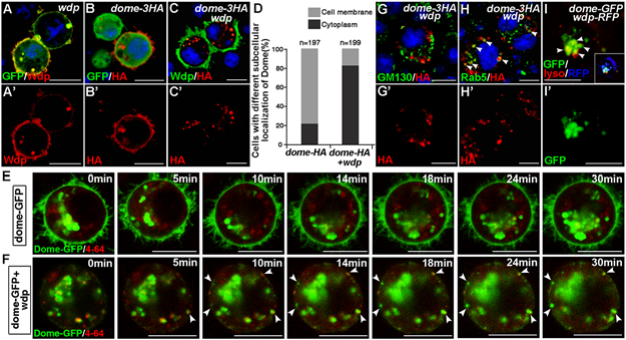
Wdp expression promotes Dome endocytosis and alters its subcellular localization in S2 cells. (A and A’) Wdp (red, by Wdp) was localized on the cell membrane in S2 cells cotransfected with *UAS-wdp* and *GFP-GPI* vectors. (B-C’) The subcellular localization of Dome in S2 cells transfected with the indicated vectors. In S2 cells expressing *dome-HA* alone, Dome-HA was mainly localized on the cell membrane and only few punctates were detected in the cytoplasm (B and B’). However, in S2 cells cotransfected with *dome-HA* and *wdp*, the majority of Dome-HA was detected as punctate particles in the cytoplasm instead of on the cell membrane (C and C’). (D) Ratio of S2 cells with Dome localized either on the cell membrane or in the cytoplasm when they were transfected with indicated vectors. (E and F) S2 cells transfected with *dome-GFP* alone (E) or in combination with *wdp* (F) were treated with 5μg/ml endocytic dye FM4-64 for 1h. And then time-lapse imaging was performed to detect dynamics of Dome-GFP and chase its co-localization with FM 4–64 at indicated time points. Arrowheads in F indicate the newly formed endocytic vesicles containing Dome-GFP on the cell membrane. (G-H’) S2 cells expressing Dome-HA and Wdp were immunostained with GM130 (G and G’) or Rab5 antibody (H and H’) to mark cis-Golgi or early endosome respectively. Dome-HA was partially co-localized with Rab5 (H, arrowheads). However, no obvious co-localization between Dome-HA and GM130 was observed (G). (I and I’) S2 cells cotransfected with *dome-GFP* and *wdp-RFP* were live stained with 50nM lysotracker solution for 2h. Dome-GFP was found to partially co-localize with the lysotracker as arrowheads shown in I. Square box is reduced image showing the overall expression of Dome-GFP (green), Wdp-RFP (blue) and lyso-tracker (red). Blue indicates DAPI staining in A-C and G-H. Scale bars, 10μm.

The JAK/STAT pathway is under tight control at various steps by different regulators and regulatory mechanisms. Since Wdp functions upstream of Hop and downstream of Upd, we examined the possible regulation of Wdp to the Dome receptor. Importantly, the total levels of Dome were reduced in S2 cells coexpressing Wdp (Fig 5H), implying that Wdp may affect the stability of Dome. Previous study showed that JAK/STAT signaling is negatively regulated by endocytic trafficking [59]. One possibility is that Wdp promotes Dome endocytosis for subsequent degradation. To test this, we performed the following experiments. First, when S2 cells were transfected with *Dome-HA* alone, Dome was mainly localized on the cell membrane, with a few punctates detected in the cytoplasm (Fig 6B, 6B’ and 6D). However, when co-expressed with Wdp, the majority of Dome was present in the cytoplasm as vesicle-like punctates (Fig 6C, 6C’ and 6D), implying the endocytosis of Dome is enhanced. To further determine whether Wdp could promote Dome endocytosis, we carried out time-lapse imaging experiments. After the live S2 cells expressing Dome-GFP were incubated with endocytic dye FM 4–64 at room temperature for 1h, we examined the dynamics of Dome-GFP and chased its co-localization with FM 4–64 at different time points. As shown in Fig 6E, in the absence of Wdp the majority of Dome-GFP was localized on the cell membrane and little co-localization with FM 4–64 was detected. When cotransfected with *wdp*, Dome-GFP was mainly observed as intracellular particles, which were partially co-localized with FM 4–64 (see arrowheads in Fig 6F). Furthermore, we observed newly formed Dome-GFP endocytic vesicles trafficking from the cell membrane (S1 Movie). All of these tissue culture data based on the overexpressed Wdp suggest that Wdp can promote Dome internalization.

We further examined the co-localization of Dome with various vesicular markers in S2 cells. Little co-localization was observed between intracellular Dome and the cis-Golgi apparatus as marked by GM130 (Fig 6G and 6G’), implying the presence of intracellular Dome-GFP was not due to defects in exocytosis. However, a large amount of Dome intracellular particles were co-localized with the early endosome marker Rab5 (Fig 6H and 6H’). In addition, high levels of Dome-GFP were present in the lysosomes as labeled by lyso-tracker in live cells (Fig 6I and 6I’). Moreover, the appearance of Dome intracellular punctates observed in Wdp-coexpressing cells was partially suppressed by Rab5 dsRNA treatment (S7 Fig), indicating that the accumulation of Dome intracellular punctates was a result of Rab5-mediated endocytosis. Interestingly, the subcellular localization of other membrane proteins such as CD8-mRFP or GFP-GPI was not affected when coexpressed with Wdp (S8 Fig), suggesting that Wdp specifically promotes Dome endocytosis.

**Fig 7:**
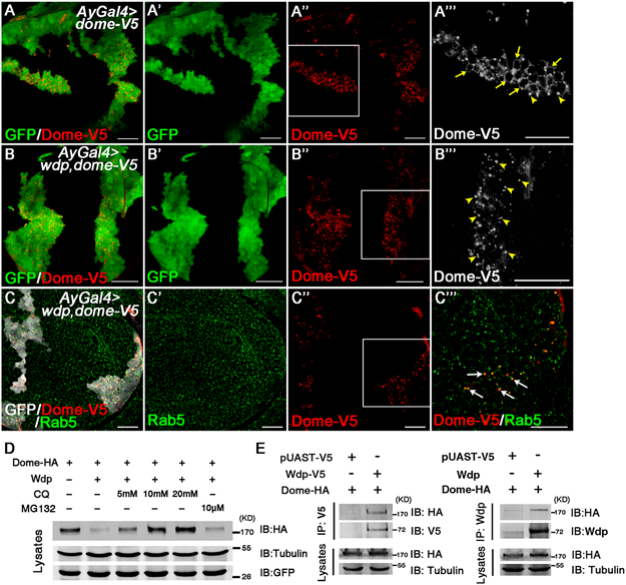
Wdp interacts with Dome to promote its internalization for lysosomal degradation. (A-A‴) In wing discs bearing GFP positively marked clones overexpressing Dome-V5 (*Act>y+>Gal4*, *UAS-GFP*, *UAS-dome-V5*), Dome-V5 was mainly localized on the cell membrane (yellow arrows) despite some intracellular punctates (yellow arrowheads). A‴ is the enlarged image of the position labeled by square box in A”. (B-B‴) Coexpression with Wdp alters the subcellular localization of Dome-V5. In wing discs bearing GFP positively marked clones expressing Dome-V5 together with Wdp (*Act>y+>Gal4*, *UAS-GFP*, *UAS-dome-V5*, *UAS-wdp*), Dome-V5 was depleted from cell membrane but detected as cytoplasmic punctate structures (yellow arrowheads). B‴ is the enlarged image of the position labeled by square box in B”. (C-C‴) The intracellular particles of Dome-V5 in the presence of Wdp were partially colocalized with early endosomes marked by Rab5 staining (white arrows). C‴ is the enlarged image of the position labeled by square box in C”. (D) To detect whether the internalized Dome was degraded, different concentrations of Chloroquine (5, 10 or 20mM) or 10 uM MG132 were used to treat S2 cells transfected with UAS-*dome-HA* and UAS-*wdp*. Then cell lysates were analyzed by western blotting with the indicated antibodies. (E) Wdp interacts with Dome in transfected S2 cells. HA-tagged *dome*, V5-tagged *wdp* (or no tagged *wdp)*, or pUAST-V5 were transfected individually or together into S2 cells. Cell lysates were immunoprecipitated and analyzed by western blotting with the antibodies indicated. All the wing discs shown here are oriented anterior left, dorsal up. Scale bars, 20μm.

We also examined whether Wdp functions similarly *in vivo*. We generated flip-out clones overexpressing Dome alone or along with Wdp in the wing and eye imaginal discs. Consistent with previous reports [59–61], Dome-V5, as a transmembrane receptor, was mainly localized on the cell membrane, and also formed some intracellular punctate structures which could correspond to endocytic vesicles (Fig 7A–7A‴). Importantly, coexpression with Wdp caused a significant change in the subcellular localization of Dome (Fig 7B–7B‴). In the presence of Wdp, Dome was totally disappeared from the cell membrane, but was found as intracellular punctates (Fig 7B–7B‴), where they partially colocalized with the early endosome marker Rab5 (Fig 7C–7C‴). In addition, we also observed the same phenomena in the eye discs (S9 Fig). Therefore, these data suggest that enhanced Wdp expression could promote Dome internalization in the wing and eye discs.

To further determine whether Dome is degraded in the lysosomes after being internalized, S2 cells were treated with Chloroquine (CQ), a lysosomal inhibitor. Interestingly, we found the reduction of Dome levels caused by Wdp co-expression was restored upon Chloroquine treatment but not upon MG132 treatment, suggesting that the internalized Dome is undergoing lysosomal degradation rather than proteasome degradation (Fig 7D). This result is in agreement with the recently published paper showing that Dome undergoes lysosomal degradation [62]. Taken together, our data indicate that Wdp functions to promote Dome endocytosis through the endosomes, and subsequently to the lysosomes for degradation.

We then investigated whether Wdp interacts with Dome to promote its endocytosis. We transfected HA-tagged *Dome* and *wdp* (or V5-tagged *wdp)* into S2 cells and found HA-tagged Dome could co-immunoprecipitate with both Wdp and V5-tagged Wdp (Fig 7E). These data indicate that Wdp interacts with Dome in transfected cells. Taken together, our data suggest that Wdp interacts with Dome and then promote its internalization from the cell membrane into the early endosomes, and finally to the lysosomes for degradation. In this way, Wdp attenuates JAK/STAT signaling to avoid uncontrolled signaling activation.

## Discussion

In this study we have provided evidence that the LRR protein Wdp is a novel component of the JAK/STAT pathway that acts in a negative feedback manner to modulate JAK/STAT signaling activity and control intestinal homeostasis. Our *in vivo* and *in vitro* data indicate that *wdp* expression levels are positively regulated by JAK/STAT signaling. Loss of *wdp* disrupts midgut homeostasis under both physiological and damage conditions. Conversely, ectopic expression of Wdp leads to the reduction of JAK/STAT signaling activity. Mechanistically, we show that Wdp can interact with Dome, and promote Dome internalization and lysosomal degradation, thereby reducing JAK/STAT signaling activity.

### Wdp controls intestinal homeostasis through interfering with JAK/STAT signaling activity

Midgut homeostasis is tightly controlled by different signaling pathways. During tissue damage, JAK/STAT, EGFR, JNK and Hippo signaling pathways are required for ISC proliferation and midgut regeneration [26, 30, 32, 33, 46, 63–65]. On the other hand, other signaling pathways, such as BMP signaling, may negatively regulate intestinal homeostasis after injury, although there exists some controversy about the function of BMP signaling during *Drosophila* intestinal development [36–39]. However, the mechanism of how ISC activity returns to quiescence after injury remains largely unknown. Here, we demonstrate that Wdp controls intestinal homeostasis through interfering with JAK/STAT signaling activity to avoid tissue hyperplasia.

Our data indicate that loss of Wdp disrupts midgut homeostasis under normal conditions and potentiates tissue regeneration under damage conditions (Figs 2 and 3). The proliferation rate of ISCs mutant for *wdp* is increased, while the differentiation of EC and ee cells is not inhibited (Fig 3 and S3A–S3D Fig). In addition, ectopic Wdp expression suppressed the damage induced tissue regeneration. Our data further demonstrate that Wdp controls intestinal homeostasis through interfering with JAK/STAT signaling activity (Fig 4). First, Wdp acts as a JAK/STAT downstream target and its expression levels are positively regulated by JAK/STAT signaling (Fig 1 and S2 Fig). Second, Wdp functions in a negative feedback loop to modulate JAK/STAT signaling activity (Fig 4 and S4 Fig). It is interesting to note that JAK/STAT signaling is mainly activated in ISCs and EBs [26]. However, we found that Wdp expression levels seem higher in ECs compared with progenitor cells (S1A–S1D’ Fig). One explanation is that low levels of Wdp in progenitors may guarantee high levels of JAK/STAT signaling, while high levels of Wdp in ECs may serve to reduce Dome levels thereby making ECs insensitive to Upd ligands. Consistent with this view, previous work showed that Dome is mainly expressed in the progenitors but not in their progeny [26]. Moreover, we found Wdp knock down using EC specific *Myo1A* ^*ts*^ also leads to the disruption of midgut homeostasis and the presence of 10×STAT GFP in putative EC cells (S4G–S4H’ Fig), suggesting that JAK/STAT signaling is activated upon *wdp* knockdown in ECs. On the other hand, we found Wdp expression was reduced but not totally eliminated in JAK/STAT signaling deficient cells (S2 Fig), suggesting that the basal level of Wdp in intestines (especially in ECs) may also be regulated by other regulatory mechanisms or signaling pathways. Further experiments are needed to clarify this issue.

It’s important to mention that Wdp expression could be induced under injury conditions, such as DSS or bleomycin treatment (S2I Fig). Consistent with our results, two recent studies also identified *wdp* as an upregulated gene upon Ecc15 and *Pseudomonas entomophila* (*P*.*e*) infection through their microarray data respectively [44, 66]. These stress conditions are also associated with the activation of JAK/STAT signaling [26, 46]. Therefore, their findings are consistent with our view that Wdp can be induced by the JAK/STAT pathway and then restrict its signaling activity in restoring intestinal homeostasis after tissue damage.

We further demonstrated the regulation of Wdp to JAK/STAT signaling in eye discs and S2 cells. 10×STAT GFP activity was decreased in eye discs overexpressing Wdp (Fig 4A–4D’) while increased in *wdp* mutant eye discs (S4A–S4D‴ Fig). Similarly, a reduction of *10×STAT luciferase* activity was also observed in S2 cells transfected with Wdp (Fig 4N). Thus, we propose that Wdp is also likely to modulate JAK/STAT signaling activity for proper development of other tissues.

Taken together, we conclude that Wdp is involved in controlling intestinal homeostasis through interfering with JAK/STAT signaling in a negative feedback manner.

### Wdp inhibits JAK/STAT signaling through promoting Dome endocytosis

Previously, several studies have addressed the roles of endocytosis in regulating JAK/STAT signal pathway. The Noselli lab found blocking internalization led to an inhibition of JAK/STAT signaling activity [61], while the Zeidler group reported the opposite results [59]. Moreover, several recent studies demonstrate that loss of *ept/tsg101* or Rabex-5, two endocytic tumor suppressor genes, also induced JAK/STAT signaling activation and tissue overgrowth [67, 68]. Yet, the regulatory mechanism of how Dome receptors are internalized remains largely unknown. Here we demonstrate that Wdp promotes Dome endocytosis and subsequent lysosomal degradation. First, in S2 cells Wdp ectopic expression induces the formation of Dome endocytotic vesicles which were colocalized with the early endosome marker and lysosome marker (Fig 6). Second, we found Wdp expression can also promote Dome endocytosis in wing and eye imaginal discs. Furthermore, the decreased Dome levels caused by Wdp expression can be suppressed by CQ treatment (Fig 7). All of these data argue that Wdp acts to promote Dome endocytosis from the cell membrane, first into the early endosomes, and finally into the lysosomes for degradation. Previous work are mainly about Dome receptors undergo ligands induced endocytosis [59, 61], while in this work we show that Wdp is able to promote Dome internalization in a Upd independent manner. Our coimmnoprecipitation data indicate Wdp can interact with Dome (Fig 7E). Moreover, S1 Movie shows that Dome-GFP are aggregated on the cell membrane before they are internalized in the presence of Wdp. Therefore, one possible mechanism is that Wdp interacts with Dome, induces the aggregation of Dome on the cell membrane and then promotes Dome endocytosis. Further experiments are needed to define the detailed mechanism.

### A model for the role of Wdp in regulating JAK/STAT pathway during tissue damage

**Fig 8:**
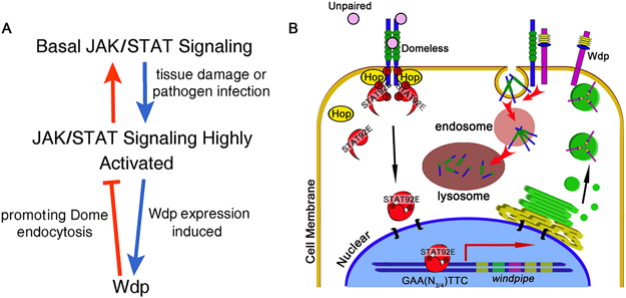
Model for the function of Wdp. During tissue damage or pathogen infection induced midgut regeneration, the JAK/STAT pathway is highly activated (A). In response to extracellular signaling, STAT92E dimers translocate into the nucleus, bind to its consensus binding sites at the genomic region of *wdp* and then promote its transcription. Newly synthesized Wdp protein is transported to the cell membrane, where it interacts with Dome and promotes Dome internalization from the cell membrane finally into the lysosomes for subsequent degradation (B). Through this negative feedback manner, Wdp restricts the signal duration and ensures JAK/STAT signaling returns to the normal levels after injury in *Drosophila* intestines.

On the basis of our findings, the following model is proposed (Fig 8A and 8B): Wdp regulates intestinal homeostasis through its modulation of JAK/STAT signaling. Under physical conditions, low levels of Wdp in progenitors are needed to maintain proper levels of JAK/STAT signaling activity, while high levels of Wdp in ECs reduce Dome levels to ensure these cells are insensitive to JAK/STAT signaling. When midgut epithelium is damaged by environmental challenges, high levels of JAK/STAT signaling activity are induced to replenish the damaged midgut. Then Wdp expression is highly induced in the intestines to reduce Dome levels, thereby switching off the overactivated JAK/STAT signaling. Through this way, ISC proliferative rate returns to normal levels to avoid tissue hyperplasia. While other mechanisms or regulators are likely to be involved in regulating intestinal homeostasis, our data suggest that Wdp is one of the key regulators in this process through interfering with JAK/STAT signaling activity.

## Methods

### Fly genetics

Information for alleles and transgenes used can be found either in FlyBase or as noted: *P{wHy}wdp* ^*DG23704*^(BL20481), *wdp* ^*1*^, *wdp* ^*2*^, *w1118*, *esg-lacZ*, *Dl-lacZ*, *Su(H)GBE-lacZ* (gift from Sarah Bray), *10×STAT GFP(II)*, *10×STAT GFP(III)*, *10×STAT DGFP*, *FRT* ^*42D*^, *FRT* ^*G13*^, *FRT* ^*82B*^, *wdp RNAi(75B)*, *UAS-upd/cyo*, *UAS-STAT*, *esgGal4-gal80* ^*ts*^ *-UAS-GFP/cyo* (gift from Norbert Perrimon), *Su(H)GBE-LacZ; esgGal4-gal80* ^*ts*^ *-UAS-GFP*, *UAS-wdp(36B)/cyo*, *UAS-wdp(86F)/Tm6B*, *UAS-STAT RNAi* (BL33637), *UAS-domeless RNAi* (BL34618), *UAS-hop 3w* (gift from Rongwen Xi), *UAS-dome-V5*, *UAS-RFP*, *EnGal4*, *mirrorGal4*, *Myo1A Gal4;tub Gal80* ^*ts*^ (gift from Steven. Hou), *Notch* ^*264-39*^, *STAT92E* ^*06346*^ *–FRT* ^*82B*^ (gift from Rongwen Xi), *UAS-Notch RNAi; STAT92E* ^*06346*^ *-FRT* ^*82B*^ (gift from Rongwen Xi). The genotypes of all flies used in this paper can be found in S1 Text.

### Constructs

*pUAST-wdp*, *wdp-V5* and *wdp-RFP* were constructed by cloning the *wdp* cDNA into *pUAST-attB*, *pUAST-attB-V5* and *pUAST-RFP* vectors respectively. *Dome-V5* and *hop-V5* were constructed by insertion of the coding region, from transgenic lines *UAS-Dome* (a gift from S. Hou) and *UAS-Hop3w* (a gift from Rongwen Xi), into *pUAST-V5-attB* vector. Dome was excised from *pUAST-dome-V5* and inserted in *pUAST-3HA* or *pUAST-GFP* to generate *pUAST-dome-3HA* or *pUAST-dome-GFP* vectors respectively. *pAC5*.*1-upd-V5* was made by cloning *upd* cDNA into *pAC5*.*1-V5* vectors. *UAS-wdp RNAi* was made by cloning annealed oligos ctagcagtAGAGGAGAGCGATGTTAGACCtagttatattcaagcataGGTCTAACATCGCTCTCCTCTgcg and aattcgcAGAGGAGAGCGATGTTAGACCtatgcttgaatataactaGGTCTAACATCGCTCTCCTCTactg into EcoRI/ NheIsites of pWalium20 vector[69] and was confirmed to be functional (S1H and S1I Fig). *10×STAT luciferase* vector was generated by subcloning *firefly luciferase* gene into *10×STAT Gal4* vector. To determine the binding sites of STAT92E in *wdp* genomic regions, we generated luciferase vectors containing putative binding regions based on the ChIP results. Primers used for constructing luciferase vectors can be found in S1 Text.

### Wdp antibody generation

We generated polyclonal antibody specific for *Drosophila* Wdp protein by choosing the hydrophilic polypeptides 480-550aa and 591-661aa as the antigen. GST-tagged Wdp antigen was expressed in E. coli BL21 (DE3) and purified with GST affinity chromatography. Using this antigen, we generated and further separated mouse polyclonal antibody of *Drosophila* Wdp.

### MARCM clone

MARCM clones in the adult midguts were induced by heat-shocking 3–4 day-old females for 75 min at 37°C. Adult guts were dissected and examined 6 days after clone induction.

### Flip-out clone

For Flip-out clones in adult midguts, crosses were set up and cultured at 25°C. Flies were heat-shocked at 37°C for 75 minutes 3 days after eclosion and then dissected 6 days later. For Flip-out clones in wing or eye discs, crosses were kept at 25°C. Larvae were heat-shocked for 90 minutes at 37°C 48 hours after egg deposition and dissected at late 3rd instar larva stage.

### Feeding experiments

Female adult flies at age 3 or 4 days were used to perform feeding experiments. Flies were cultured in an empty vial containing chromatography paper wet with 3% dextran sulfate sodium (MP Biomedicals) or 25μg/mL bleomycin (Sigma) dissolved in 5% glucose solution with heat inactivated yeast for 4 days at 29°C.

### Antibodies used for immunostaining, immunoprecipitation, and western blotting

Fixation and antibody staining in imaginal discs were performed as described [70]. Fixation and antibody staining in cultured cells were performed as described [71]. Fixation and antibody staining in midguts were performed as described [37]. Primary antibodies used for the immunostaining were: mouse anti-Wdp (1:1000), chicken anti-lacZ (Abcam, 1:1000), mouse anti-Dl (DSHB, 1:50), mouse anti-Pros (DSHB, 1:200), rabbit anti-PH3 (Millipore, 1: 2000), mouse anti Brdu (DSHB, 1:200), rabbit anti Pdm1(1:1000, gift from Xiaohang Yang), mouse anti-V5 (Invitrogen, 1:3000), mouse anti-HA (Abmart, 1:500), rabbit anti-GM130 (Abcam, 1:200), rabbit anti-Rab5 (Abcam, 1:200), Gp anti-Sens (1:200), rabbit anti-Sal (1:100), and Rat anti-Ci (DSHB, 1:5). The primary antibodies were detected by fluorescent-conjugated secondary antibodies from Jackson ImmunoResearch Laboratories, Inc. The primary antibodies used for IP and western blot were: rabbit anti-V5 (Sigma, 1:1000), rabbit anti-HA (Santa Cruz, 1:1000), mouse anti-Wdp (1:500), rabbit anti-GFP (Abmart, 1:1000) and mouse anti-tubulin (Abmart, 1:1000).

### Brdu incorporation

Adult flies with MARCM clones were reared on standard corn meal food with 0.2mg/ml BrdU (Sigma) at 25°C for 4 days before dissection. Then midguts were treated with 3M HCl at 37°C for 30 min, and the reaction was stopped by washing with PBT twice.

### RT-qPCR

RNA was extracted from 20 intestines of female adults using RNA pre pure kit (TIANGEN) and complementary DNA (cDNA) was synthesized with the M-MLV Reverse transcriptase (Promega). qPCR was performed using GoTaq qPCR Master Mix kit (Promega) on CFX96 Real-time PCR system(Bio-Rad). Experiments were performed in 3 biological independent replicates, each also contained 3 repeats. All the results are shown as Mean±SD of the biological replicates. Ribosomal gene RpL11 was used as normalization control. Primers used for qPCR are listed in S1 Text.

### ChIP-Seq

The identification of STAT92E target genes in adult intestines was carried out through ChIP assay and ChIP-high throughput sequencing technique. JAK/STAT signaling was activated using *esg* ^*ts*^ to overexpress *Upd* and *STAT92E* at 29°C for 10 days. Then about 400 adult intestines were dissected and cross-linked with 1% formaldehyde for 15 minutes. After washing process to remove the formaldehyde, intestinal tissue was lysed with RIPA buffer which contains 1% SDS on ice for 30 minutes. The sonication of chromatin was performed using the Covaris (AFA) system with 3% power output for 5 minutes each on 100μl lysate. STAT92E-bound chromatin fragments were enriched by immunoprecipitation with mouse raised STAT92E antibody. Most chromatin fragments resulting from sonication occurred between 200 and 400 bp. The process of dilution, antibody incubation, protein G pull down, beads washing, DNA complex elution, de-link, RNAse A / proteinase K digestion and DNA extraction are all performed according to standard protocols. The high throughput sequencing process was carried out using the Illumina solexa system.

### Cell culture, transfection, coimmunoprecipitation and western blotting

*Drosophila* S2 cells were maintained at 25°C in HyQ SFX-insect cell culture medium. All transfection experiments were carried out using Effectene Transfection Reagent (QIAGEN).

For Wdp and Dome interaction experiments, S2 cells were transfected in 60mm dishes with 200ng *Arm-Gal4*, 200ng *pUAST-dome-HA* and 200ng *pUAST-V5* control vector, or *pUAST-wdp* (with or without V5 tag). Then S2 cells were lysed in 200μl RIPA buffer without SDS on ice for 30 minutes. RIPA buffer includes 50 mM Tris-HCl (pH 7.8), 150 mM NaCl, 5 mM EDTA (pH 8.0), 0.5% Triton X-100, 0.5% NP-40, 0.5%DOC, complete protease inhibitor cocktail tablets (Roche), and phosphatase inhibitor cocktail tablets (Roche). After centrifugation, the suspension of lysates was added with antibody and incubated for 3h at 4°C, and then added with BSA blocked protein G beads and rotated overnight at 4°C. The immunocomplexes were collected by centrifugation and washed with 1 ml of RIPA buffer three times.

For lysosome or proteasome inhibition assay, S2 cells were treated with 5, 10 or 20 mM Chloroquine (Sigma-Aldrich) or 10 uM MG132 (Sigma-Aldrich) respectively for 24 h before harvesting.

For western blotting, immunoprecipitated proteins were separated in SDS-PAGE and then blotted onto PVDF membranes. The membranes were stained with primary antibody overnight at 4°C, as anti-V5, anti-HA, anti-Wdp to detect interaction between Wdp and Dome. Antibody HA was used to examine the effects of Wdp on Dome levels. Followed by washing, PVDF membranes were incubated with secondary antibodies carrying infrared fluorophore, and then analyzed using Odyssey system (GENE).

### Luciferase assay

S2 cells were seeded in 24-wells plate. Cells in each well were transfected with 5ng *Renilla-luciferase*, 30ng *10×STAT-luciferase* reporter (or other reporters) and 30 ng other vectors as shown in figures. After 12h, cells were mixed with Upd transfected cells. After an additional 48h, S2 cells were washed with PBS and then lysed using Passive Lysis Buffer (Promega). Firefly-luciferase and Renilla-luciferase activity were detected using GLOMA Multi Detection System (Promega). All the results are from twice independent experiments each containing 3 repeats.

### Live cell imaging

For labeling of endocytic vesicles, S2 cells were treated with 5μg/ml FM4-64 (Molecular Probes, Inc.) at 25°C for 1h. S2 cells were then washed twice with medium and then incubated at 25°C for another 1h. Then 200ul of cell suspension was applied to a microscope slide. Images were captured by a Zeiss LSM780 inverted confocal microscope and movies were made from time-lapse images using Corel Video Studio X4. For labeling of lysosomes, S2 cells were incubated with cell culture containing lyso-tracker (Invitrogen) at a final concentration of 50 nM at 25°C for 2h.

## Supporting Information

S1 Fig The expression pattern of Wdp in different developmental processes. (A-D’) Wdp (red, by Wdp) was ubiquitously expressed in both progenitor cells and ECs in adult posterior midguts. *esg-lacZ* (A and A’), *Su(H)GBE-lacZ* (B and B’) and *Dl-lacZ* (C and C’) was used to mark progenitor cell, EBs and ISCs respectively. EC cells are labeled by large nucleus. Squared box in C’ shows the enlarged image of the position labeled by yellow arrow. (E) The generation of wdp mutants, *wdp* ^*1*^ and *wdp* ^*2*^. Schematic drawings illustrating transcribed regions (boxed), non-transcribed regions (line), coding regions (yellow filling), P-element insertion sites (triangles), and the *wdp* ^*1*^ or *wdp* ^*2*^ deletions associated with imprecise excisions of the P-element insertions in *wdp*. (F) The transcriptional levels of *wdp* were significantly reduced from *wdp* ^*1/1*^ homozygotes using RT-qPCR quantification while the neighboring gene *gp150* was not affected. Mean±SD are shown. **p<0.01. (G and G’) Wdp staining (red, by Wdp) was reduced in intestinal *wdp* ^*1*^ MARCM clones positively marked by GFP. (H and I) Wdp expression (red, by Wdp) was diminished upon *wdp* knockdown using *EnGal4* in the posterior compartment of wing discs. The wing discs here are oriented dorsal-up, anterior-left. (J) Wdp seems ubiquitously expressed in eye imaginal disc of 3^rd^ instar larva. Blue indicates DAPI staining in A-G. Scale bars, 20μm.
(TIF)

S2 Fig Wdp expression levels were reduced but not totally eliminated in JAK/STAT deficient progenitor cells. (A-D’) Wdp expression (red, by Wdp) in intestinal Flip-out clones with indicated genotypes at 29°C for 8 days (A-C’) or 14 days (D and D’). In control clones (A and A’), there were no obvious difference of Wdp expression levels between GFP+ (arrow) and GFP- cells (arrowhead). In Flip-out clones knocking down Dome or STAT (arrows in B-D’), Wdp expression levels were reduced compared with surrounding wildtype cells (arrowheads in B-D’). (E-H’) Wdp expression (red, by Wdp) in intestinal MARCM clones with indicated genotypes at 25°C for 7 days. In *FRT* ^*82B*^ control MARCM clones (E and E’), Wdp was uniformly expressed between GFP+ clone cells (arrow in E’) and GFP- cells (arrowhead in E’). However, Wdp expression was reduced in *STAT92E* ^*06346*^ clone cells (arrows in F’) compared with surrounding WT cells (arrowheads in F’). In addition, we generated *Notch* ^*264-39*^ mutant clones and detected Wdp expression mainly on the plasma membrane of ISC clusters (G and G’). As shown in G’, Wdp was also uniformly expressed between *Notch* ^*264-39*^ clones (arrow in G’) and GFP- cells (arrowheads in G’). In STAT92E mutant clone cells with simultaneous Notch RNAi, Wdp expression levels (arrows in H’) were reduced compared with Notch mutant clones (arrows in G’). Furthermore, Wdp expression was reduced in clone cells (arrows in H’) compared with surrounding *WT* cells (arrowheads in H’). (I) The mRNA levels of *wdp* were increased under damage conditions using RT-qPCR quantification. *w1118* flies aged at 3–4 days were treated with 3% DSS or 25ug/ml bleomycin at 29°C for 4 days. Mean ± SD are shown. **p<0.01. Blue indicates DAPI staining in A-H. Scale bars, 20μm.
(TIF)

S3 Fig Loss of wdp leads to the disruption of midgut homeostasis. (A and B) Compared with controls (A), the number of ISC (red, by Dl) was increased in *wdp* ^*1/1*^ homozygotes at 25°C for 7 days (B). Besides, EBs were still able to differentiate into ees (red, by Pros) or large nuclei ECs in the absence of *wdp*. (C and D) EC differentiation indicated by Pdm1 staining was not inhibited in *wdp* ^*1/1*^ homozygotes. (E-G) Compared with controls (E), the number of *esg-lacZ* positive cells was increased in *wdp* ^*1/2*^ trans-heterozygotes (F). G shows the quantification of the relative number of *esg-lacZ* positive cells. Mean±SD are shown. n = 8–10 intestines. **p<0.01. Blue indicates DAPI staining in A-F. Scale bars, 20μm.
(TIF)

S4 Fig Loss of Wdp leads to the upregulation of JAK/STAT signaling activity. (A and B) Compared with controls (A), the activity and the expression regions of 10×STAT GFP were enhanced in the eye discs of *wdp* ^*1/1*^ early 3^rd^ instar larva (B). The expression regions of 10×STAT GFP are indicated by white double-headed arrows. (C) Quantification of the expression region of 10×STAT GFP in *WT* and *wdp* ^*1/1*^ homozygous early 3^rd^ instar eye discs. Mean±SD are shown. n = 6–9. **p<0.01. (D-D‴) The expression region of 10×STAT GFP was enlarged in the 3^rd^ instar eye discs upon *wdp* knockdown using *mirrorGal4*. CD8-mRFP was used to mark the dorsal compartment. Double headed arrows in D‴ show the expression region of 10×STAT GFP in the dorsal and ventral part. (E-F’) The activity of unstable 10×STAT DGFP was obviously increased in *wdp* ^*1/1*^ intestines (F and F’) compared with controls (E and E’). Moreover, 10×STAT DGFP was no longer restricted in small progenitor cells but also appeared in large ECs (arrows in F’). Figures E-F’ are taken using the same laser intensity. (G-H’) Compared with controls (G and G’), Wdp knock down in ECs using *Myo1A* ^*ts*^ led to the disruption of intestinal homeostasis (H and H’). Besides, 10×STAT GFP also appeared in large putative EC cells (arrows in H’). Blue indicates DAPI staining in E-H’. Scale bars, 20μm.
(TIF)

S5 Fig Wdp expression has no obvious effects on Wingless, Dpp or Hedgehog signaling. Wing discs bearing flip-out clones expressing *wdp* were immunostained with various antibodies to detect whether other signaling pathways were affected. Sens for Wingless signaling, Sal for Dpp signaling, Ci for Hedgehog signaling. The expression levels of Sens (A’), Sal (B’) or Ci (C’) were not altered in *wdp* expressing clones marked by the presence of GFP and overabundance of Wdp expression. All the wing discs shown here are oriented anterior right, dorsal down. Scale bars, 20μm.
(TIF)

S6 Fig Upd expression induced upregulation of JAK/STAT signaling could be partially suppressed by Wdp expression in wing discs in a cell autonomous manner. (A and A’) 10×STAT GFP in the control flip-out clones marked by RFP expression and dotted line. 10×STAT GFP was expressed surrounding the wing margin of 3^rd^ instar wing discs. (B and B’) 10×STAT GFP was ectopically expressed in the wing pouch containing flip-out clones expressing Upd in 3^rd^ instar wing discs (white arrow). Besides, the JAK/STAT signaling activity in the RFP- cells adjacent the RFP+ clones could also be induced non-autonomously (yellow arrow). (C and C’) The upregulation of 10×STAT GFP caused by Upd expression in the wing pouch could be partially suppressed by simultaneous Wdp expression (white arrow). However, the activated JAK/STAT signaling in the RFP- cells adjacent the RFP+ clones due to the diffusion of Upd couldn’t be suppressed (yellow arrow).
(TIF)

S7 Fig Rab5 dsRNA treatment could partially suppress the enhanced endocytosis of Dome-GFP caused by Wdp expression. (A-C’) S2 cotransfected with *dome-GFP* and *wdp* were treated with 10μg control *lacZ dsRNA*, *Rab5 dsRNA-1*, or *Rab5 dsRNA-2* respectively for 5 days. In the presence of Wdp, Dome-GFP was mainly present as intracellular particles under control *lacZ dsRNA* treatment (A-A’), while the appearance of Dome punctates was partially suppressed by *Rab5 dsRNA* treatment (B-B’ and C-C’). (D) The transcriptional levels of *rab5* in S2 cells which were treated with different *dsRNA* (*lacZ dsRNA*, *Rab5 dsRNA-1* or *Rab5 dsRNA-2*) for 5 days. Mean±SD are shown. **p<0.01. Blue indicates DAPI staining in A, B and C. Scale bars, 5μm.
(TIF)

S8 Fig Wdp promotes Dome internalization without affecting other membrane molecules. In live S2 cells cotransfected with *dome-GFP* and *wdp-RFP* vectors, the majority of Dome-GFP was localized as intracellular punctate structures (A and A’). However, the subcellular localization of other membrane proteins such as CD8-mRFP (B and B”) or GFP-GPI (C and C’) was not affected when coexpressed with Wdp, indicating that Wdp promotes Dome internalization without affecting the subcellular localization of other membrane molecules. Scale bars, 5μm.
(TIF)

S9 Fig Wdp expression promotes the endocytosis of Dome in eye imaginal discs. (A-A‴) In eye discs bearing GFP positively marked clones overexpressing Dome-V5 (*Act>y+>Gal4*, *UAS-GFP*, *UAS-dome-V5*), Dome-V5 was mainly localized on the cell membrane (yellow arrows) despite some intracellular punctate structures (yellow arrowheads). A‴ is the enlarged image of the position labeled by square box in A”. (B-B‴) In eye discs bearing GFP positively marked clones expressing Dome-V5 together with Wdp (*Act>y+>Gal4*, *UAS-GFP*, *UAS-dome-V5*, *UAS-wdp*), Dome-V5 was depleted from cell membrane but detected as cytoplasmic particles (B”), which were partially colocalized with early endosome marker Rab5 (B‴, white arrows). B‴ is the enlarged image of the position labeled by square box in B”. All the eye discs shown here are oriented posterior right. Scale bars, 20μm.
(TIF)

S1 Table Partial JAK/STAT targets identified from ChIP experiments with adult gut tissues. In this ChIP assay, we totally got 1487 peaks with p-value<0.01. The above table shows partial putative JAK/STAT downstream targets. Some of them have previously been reported as potential targets or components of the JAK/STAT pathway through microarray or RNAi screening methods. The binding sites of STAT92E around the ChIP peaks (±500bp) include **TTC**NN**GAA**, **TTC**NNN**GAA** and **TTC**NNNN**GAA**. The full ChIP-Seq data can be found in the GEO database with the accession number GSE67346.
(DOC)

S1 Movie Wdp expression promotes Dome-GFP trafficking from the cell membrane into the cytoplasm in live S2 cells. (Part 1–3) The dynamics of Dome-GFP in live S2 cells co-transfected with *dome-GFP* and *wdp*. Dome-GFP was mainly detected as intracellular punctuates. In Part 1, the arrowheads marked by A, B, C, D and E show the newly formed endocytic vesicles trafficking Dome-GFP from the cell membrane. Part 2 is the enlarged movie of the endocytic vesicles marked by B and C. Part 3 shows the co-localization of Dome-GFP with endocytic dye FM4-64.(Part 4–5) The dynamics of Dome-GFP in live S2 cells transfected with *dome-GFP* alone. Dome-GFP was mainly present on the cell membrane and no newly formed vesicles trafficking Dome-GFP from the cell membrane were observed (Part 4). In addition, no obvious colocalization of Dome-GFP with FM4-64 was detected in Part 5.
(AVI)

S1 Text Supplemental experimental procedures. Genotypes of flies used in Figs 1–8 and S1–S9 Figs are listed, followed by the information of primers used for RT-qPCR, constructing luciferase vectors and Rab5 dsRNA synthesis. In addition, the protocol of Rab5 dsRNA synthesis is also included.
(DOC)
