# Supplementary figures and images for: Windpipe Controls Drosophila Intestinal Homeostasis by Regulating JAK/STAT Pathway via Promoting Receptor Endocytosis and Lysosomal Degradation
Source: PLoS Genet. 2015 Apr 29;11(4):e1005180. doi: 10.1371/journal.pgen.1005180 (PMC4414558; doi:10.1371/journal.pgen.1005180)

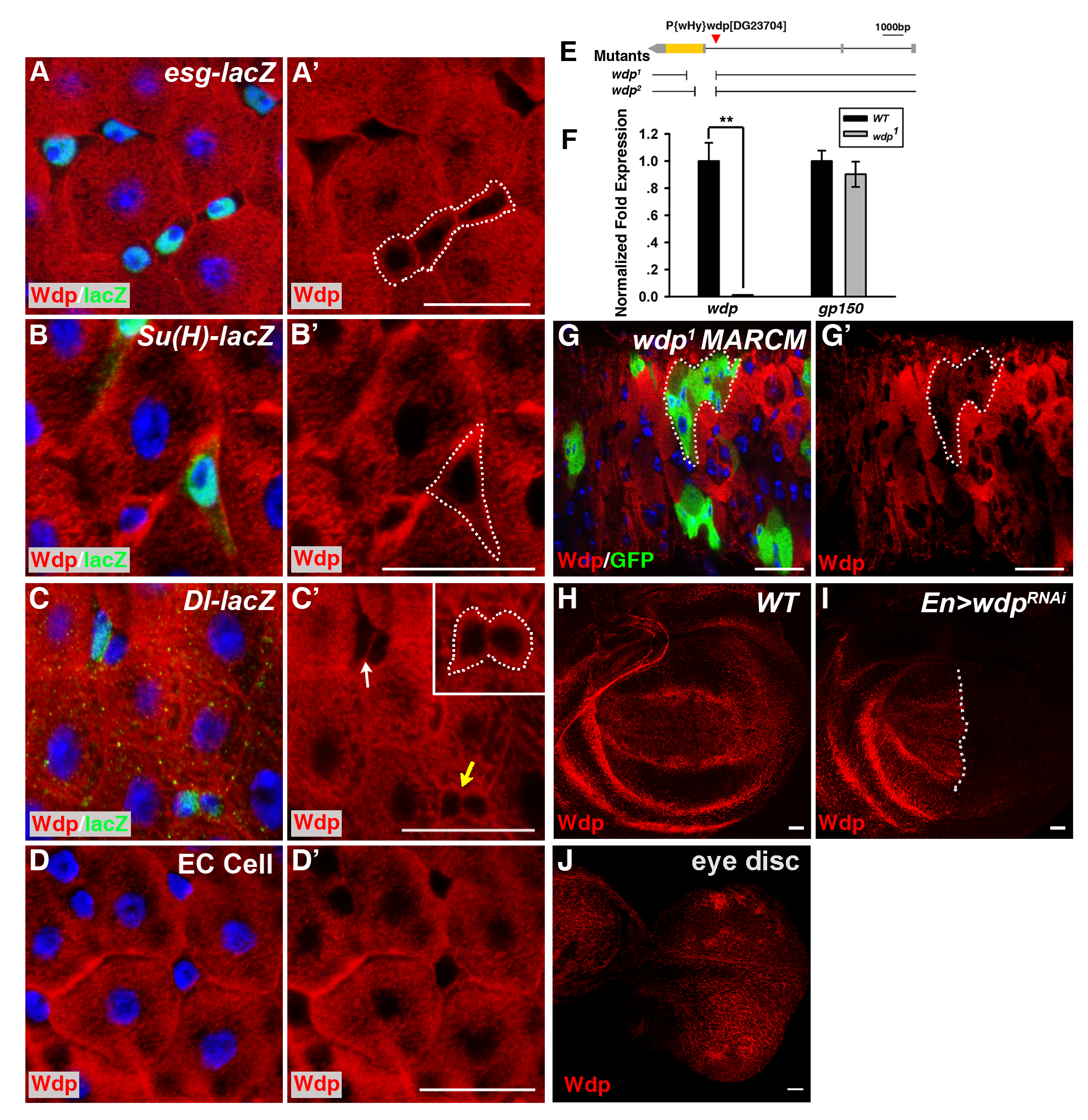

Supplement: S1 Fig — (A-D’) Wdp (red, by Wdp) was ubiquitously expressed in both progenitor cells and ECs in adult posterior midguts. esg-lacZ (A and A’), Su(H)GBE-lacZ (B and B’) and Dl-lacZ (C and C’) was used to mark progenitor cell, EBs and ISCs respectively. EC cells are labeled by large nucleus. Squared box in C’ shows the enlarged image of the position labeled by yellow arrow. (E) The generation of wdp mutants, wdp 1 and wdp 2. Schematic drawings illustrating transcribed regions (boxed), non-transcribed regions (line), coding regions (yellow filling), P-element insertion sites (triangles), and the wdp 1 or wdp 2 deletions associated with imprecise excisions of the P-element insertions in wdp. (F) The transcriptional levels of wdp were significantly reduced from wdp 1/1 homozygotes using RT-qPCR quantification while the neighboring gene gp150 was not affected. Mean±SD are shown. **p<0.01. (G and G’) Wdp staining (red, by Wdp) was reduced in intestinal wdp 1 MARCM clones positively marked by GFP. (H and I) Wdp expression (red, by Wdp) was diminished upon wdp knockdown using EnGal4 in the posterior compartment of wing discs. The wing discs here are oriented dorsal-up, anterior-left. (J) Wdp seems ubiquitously expressed in eye imaginal disc of 3rd instar larva. Blue indicates DAPI staining in A-G. Scale bars, 20μm. (TIF) [file pgen.1005180.s001.tif]

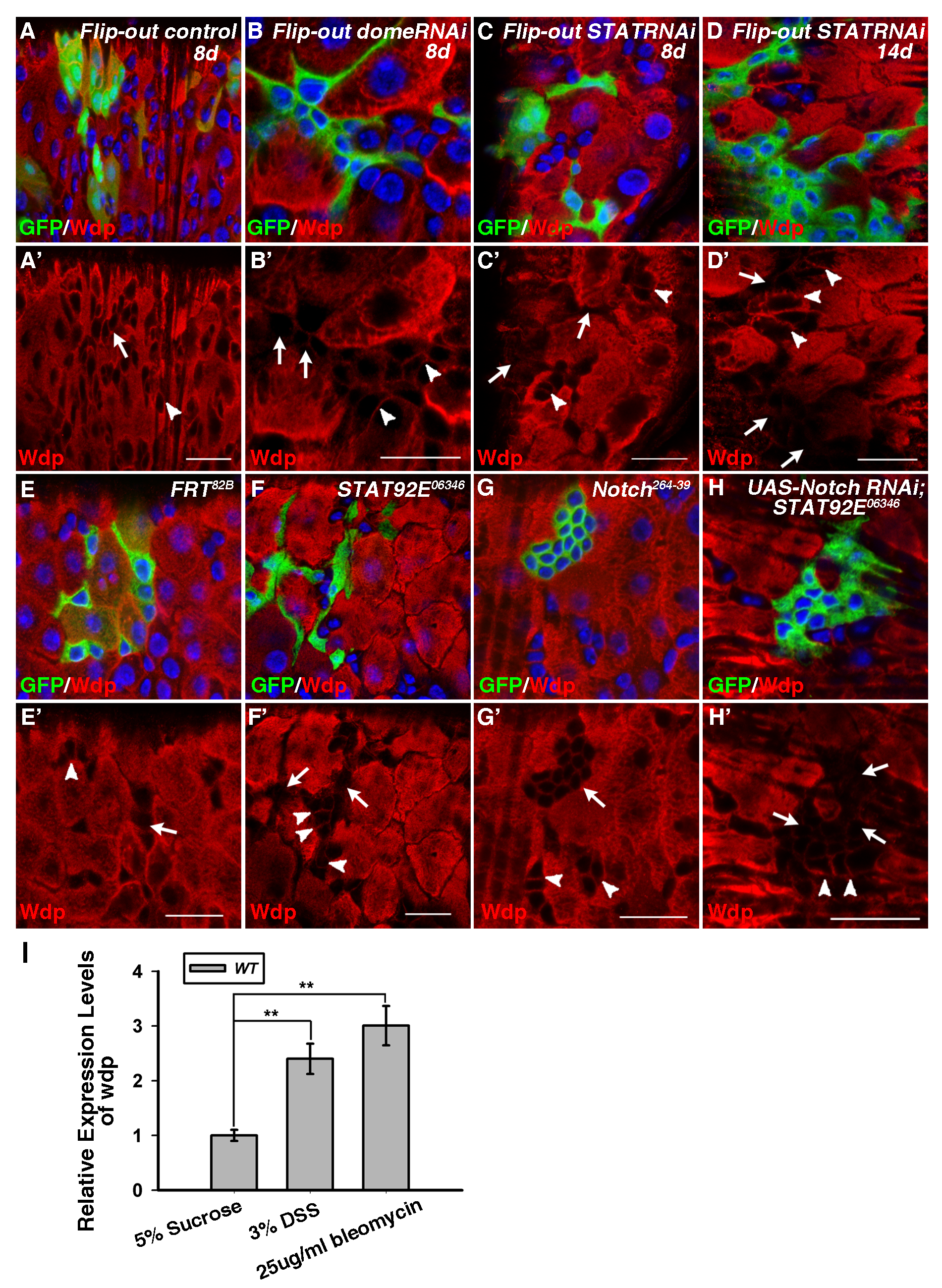

Supplement: S2 Fig — (A-D’) Wdp expression (red, by Wdp) in intestinal Flip-out clones with indicated genotypes at 29°C for 8 days (A-C’) or 14 days (D and D’). In control clones (A and A’), there were no obvious difference of Wdp expression levels between GFP+ (arrow) and GFP- cells (arrowhead). In Flip-out clones knocking down Dome or STAT (arrows in B-D’), Wdp expression levels were reduced compared with surrounding wildtype cells (arrowheads in B-D’). (E-H’) Wdp expression (red, by Wdp) in intestinal MARCM clones with indicated genotypes at 25°C for 7 days. In FRT 82B control MARCM clones (E and E’), Wdp was uniformly expressed between GFP+ clone cells (arrow in E’) and GFP- cells (arrowhead in E’). However, Wdp expression was reduced in STAT92E 06346 clone cells (arrows in F’) compared with surrounding WT cells (arrowheads in F’). In addition, we generated Notch 264-39 mutant clones and detected Wdp expression mainly on the plasma membrane of ISC clusters (G and G’). As shown in G’, Wdp was also uniformly expressed between Notch 264-39 clones (arrow in G’) and GFP- cells (arrowheads in G’). In STAT92E mutant clone cells with simultaneous Notch RNAi, Wdp expression levels (arrows in H’) were reduced compared with Notch mutant clones (arrows in G’). Furthermore, Wdp expression was reduced in clone cells (arrows in H’) compared with surrounding WT cells (arrowheads in H’). (I) The mRNA levels of wdp were increased under damage conditions using RT-qPCR quantification. w1118 flies aged at 3–4 days were treated with 3% DSS or 25ug/ml bleomycin at 29°C for 4 days. Mean ± SD are shown. **p<0.01. Blue indicates DAPI staining in A-H. Scale bars, 20μm. (TIF) [file pgen.1005180.s002.tif]

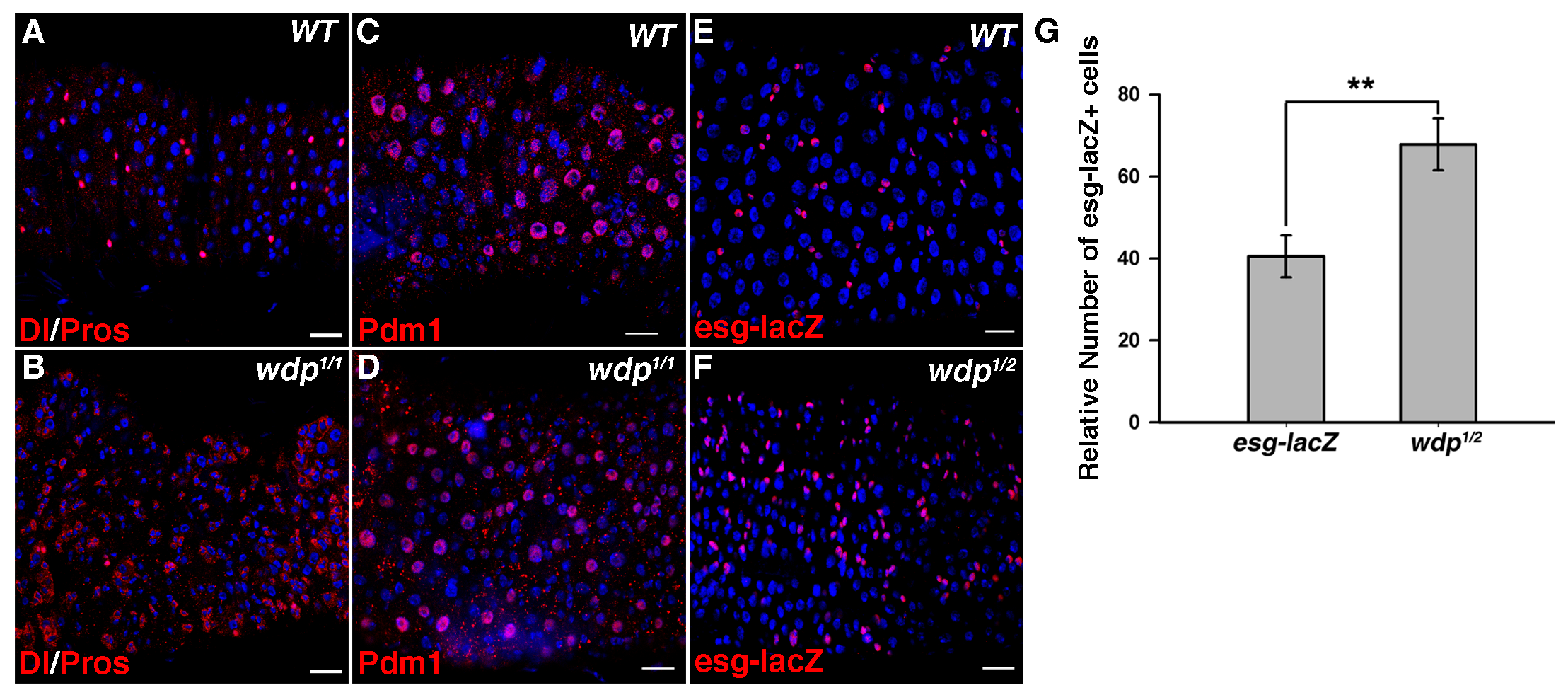

Supplement: S3 Fig — (A and B) Compared with controls (A), the number of ISC (red, by Dl) was increased in wdp 1/1 homozygotes at 25°C for 7 days (B). Besides, EBs were still able to differentiate into ees (red, by Pros) or large nuclei ECs in the absence of wdp. (C and D) EC differentiation indicated by Pdm1 staining was not inhibited in wdp 1/1 homozygotes. (E-G) Compared with controls (E), the number of esg-lacZ positive cells was increased in wdp 1/2 trans-heterozygotes (F). G shows the quantification of the relative number of esg-lacZ positive cells. Mean±SD are shown. n = 8–10 intestines. **p<0.01. Blue indicates DAPI staining in A-F. Scale bars, 20μm. (TIF) [file pgen.1005180.s003.tif]

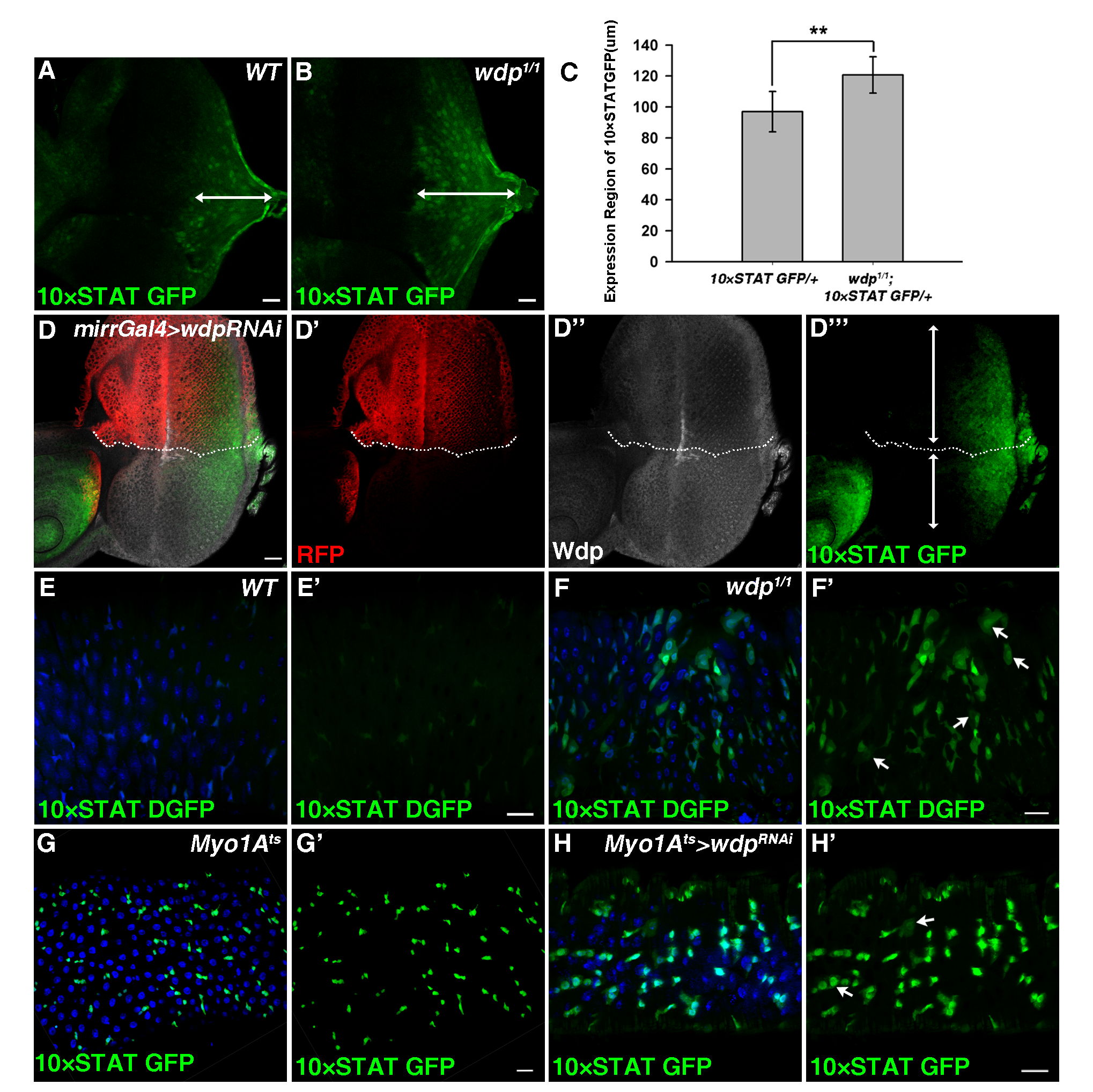

Supplement: S4 Fig — (A and B) Compared with controls (A), the activity and the expression regions of 10×STAT GFP were enhanced in the eye discs of wdp 1/1 early 3rd instar larva (B). The expression regions of 10×STAT GFP are indicated by white double-headed arrows. (C) Quantification of the expression region of 10×STAT GFP in WT and wdp 1/1 homozygous early 3rd instar eye discs. Mean±SD are shown. n = 6–9. **p<0.01. (D-D‴) The expression region of 10×STAT GFP was enlarged in the 3rd instar eye discs upon wdp knockdown using mirrorGal4. CD8-mRFP was used to mark the dorsal compartment. Double headed arrows in D‴ show the expression region of 10×STAT GFP in the dorsal and ventral part. (E-F’) The activity of unstable 10×STAT DGFP was obviously increased in wdp 1/1 intestines (F and F’) compared with controls (E and E’). Moreover, 10×STAT DGFP was no longer restricted in small progenitor cells but also appeared in large ECs (arrows in F’). Figures E-F’ are taken using the same laser intensity. (G-H’) Compared with controls (G and G’), Wdp knock down in ECs using Myo1A ts led to the disruption of intestinal homeostasis (H and H’). Besides, 10×STAT GFP also appeared in large putative EC cells (arrows in H’). Blue indicates DAPI staining in E-H’. Scale bars, 20μm. (TIF) [file pgen.1005180.s004.tif]

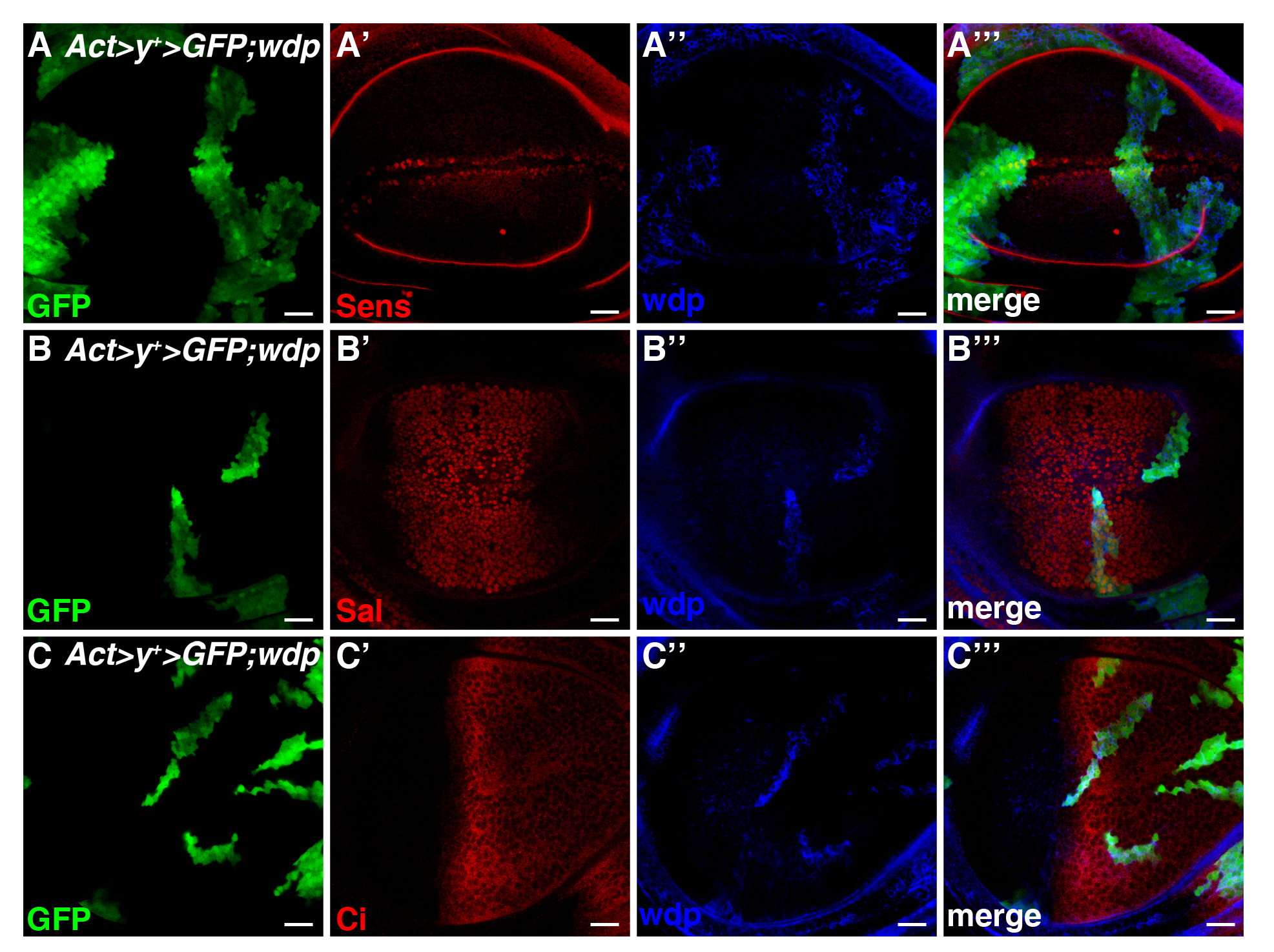

Supplement: S5 Fig — Wing discs bearing flip-out clones expressing wdp were immunostained with various antibodies to detect whether other signaling pathways were affected. Sens for Wingless signaling, Sal for Dpp signaling, Ci for Hedgehog signaling. The expression levels of Sens (A’), Sal (B’) or Ci (C’) were not altered in wdp expressing clones marked by the presence of GFP and overabundance of Wdp expression. All the wing discs shown here are oriented anterior right, dorsal down. Scale bars, 20μm. (TIF) [file pgen.1005180.s005.tif]

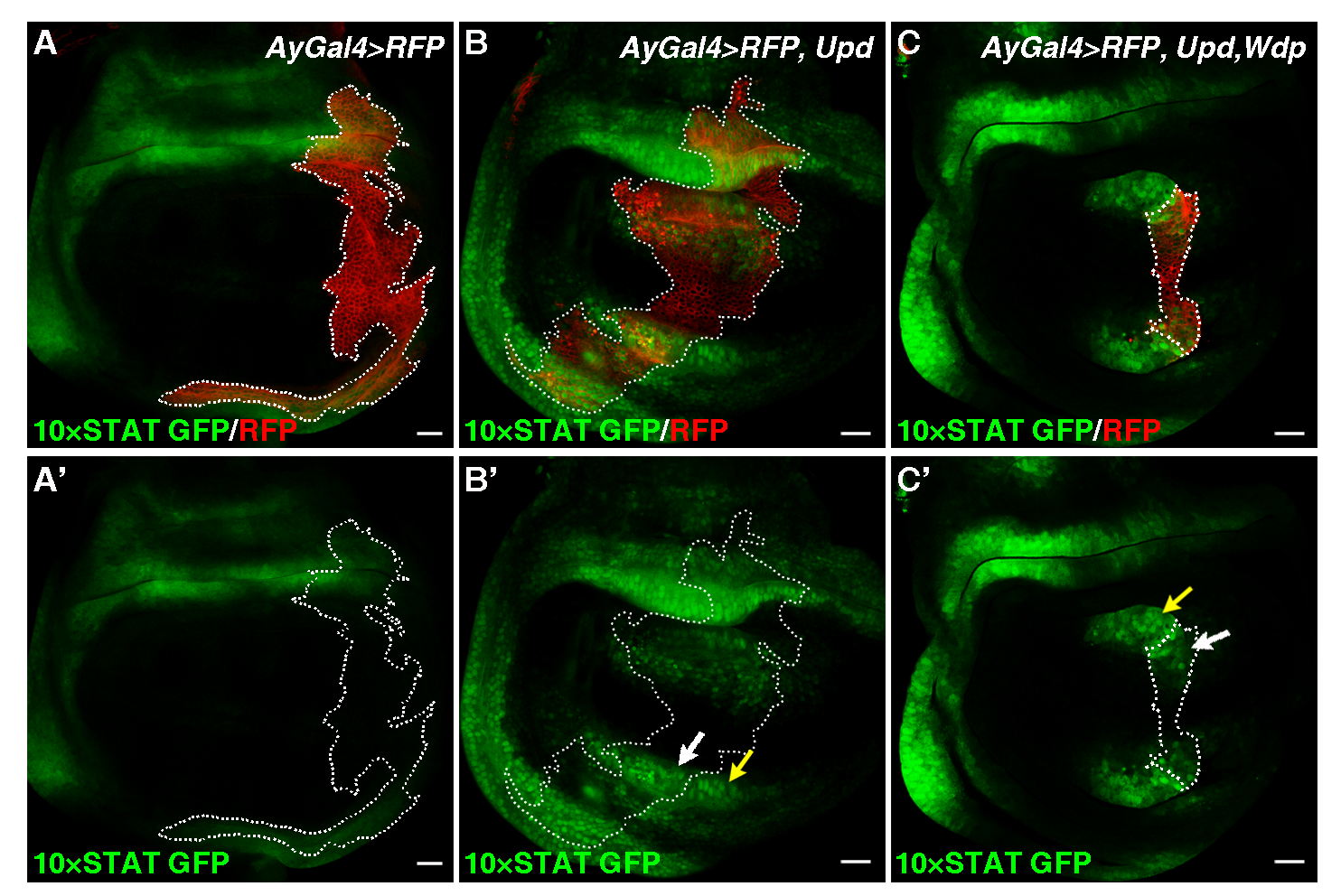

Supplement: S6 Fig — (A and A’) 10×STAT GFP in the control flip-out clones marked by RFP expression and dotted line. 10×STAT GFP was expressed surrounding the wing margin of 3rd instar wing discs. (B and B’) 10×STAT GFP was ectopically expressed in the wing pouch containing flip-out clones expressing Upd in 3rd instar wing discs (white arrow). Besides, the JAK/STAT signaling activity in the RFP- cells adjacent the RFP+ clones could also be induced non-autonomously (yellow arrow). (C and C’) The upregulation of 10×STAT GFP caused by Upd expression in the wing pouch could be partially suppressed by simultaneous Wdp expression (white arrow). However, the activated JAK/STAT signaling in the RFP- cells adjacent the RFP+ clones due to the diffusion of Upd couldn’t be suppressed (yellow arrow). (TIF) [file pgen.1005180.s006.tif]

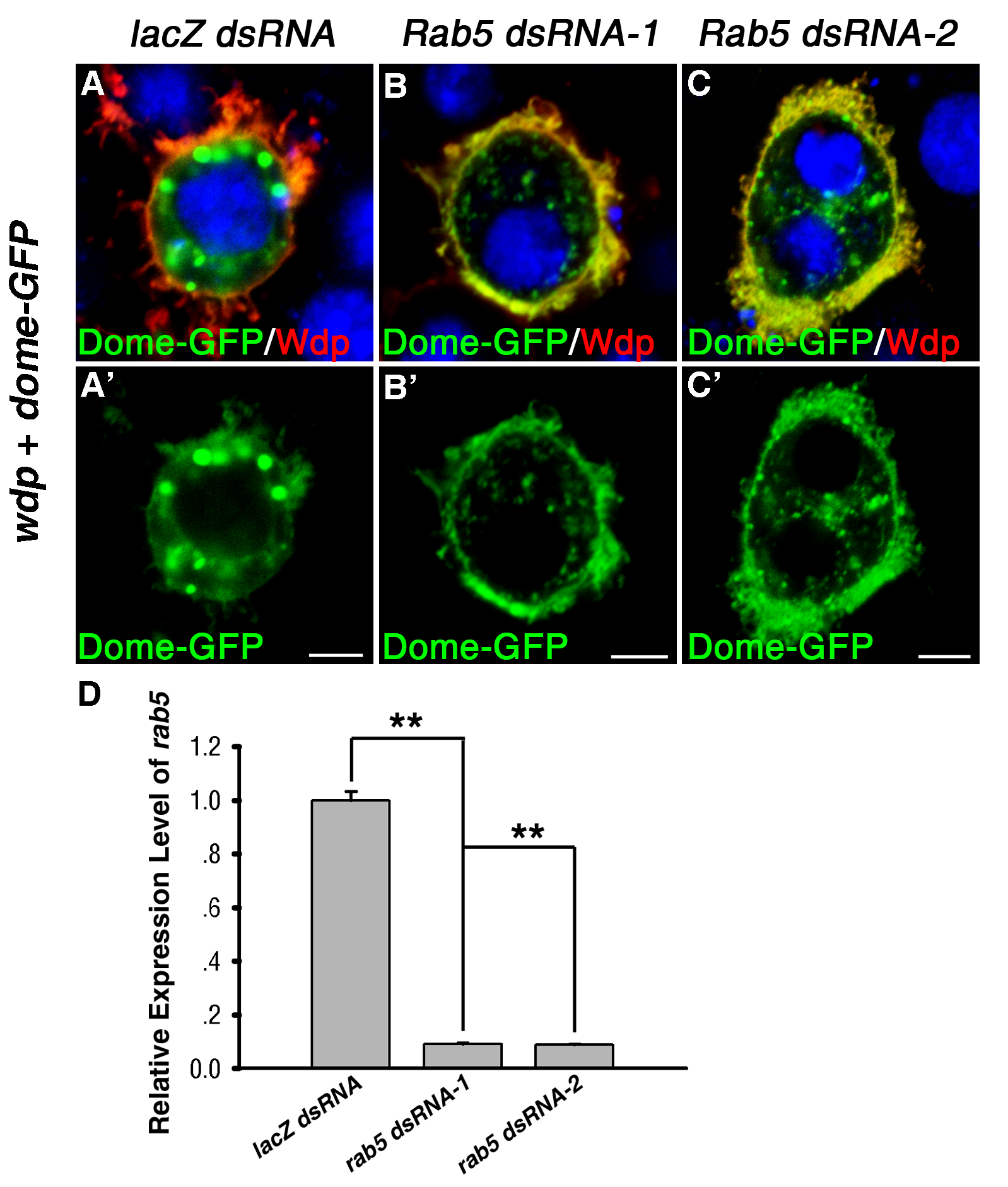

Supplement: S7 Fig — (A-C’) S2 cotransfected with dome-GFP and wdp were treated with 10μg control lacZ dsRNA, Rab5 dsRNA-1, or Rab5 dsRNA-2 respectively for 5 days. In the presence of Wdp, Dome-GFP was mainly present as intracellular particles under control lacZ dsRNA treatment (A-A’), while the appearance of Dome punctates was partially suppressed by Rab5 dsRNA treatment (B-B’ and C-C’). (D) The transcriptional levels of rab5 in S2 cells which were treated with different dsRNA (lacZ dsRNA, Rab5 dsRNA-1 or Rab5 dsRNA-2) for 5 days. Mean±SD are shown. **p<0.01. Blue indicates DAPI staining in A, B and C. Scale bars, 5μm. (TIF) [file pgen.1005180.s007.tif]

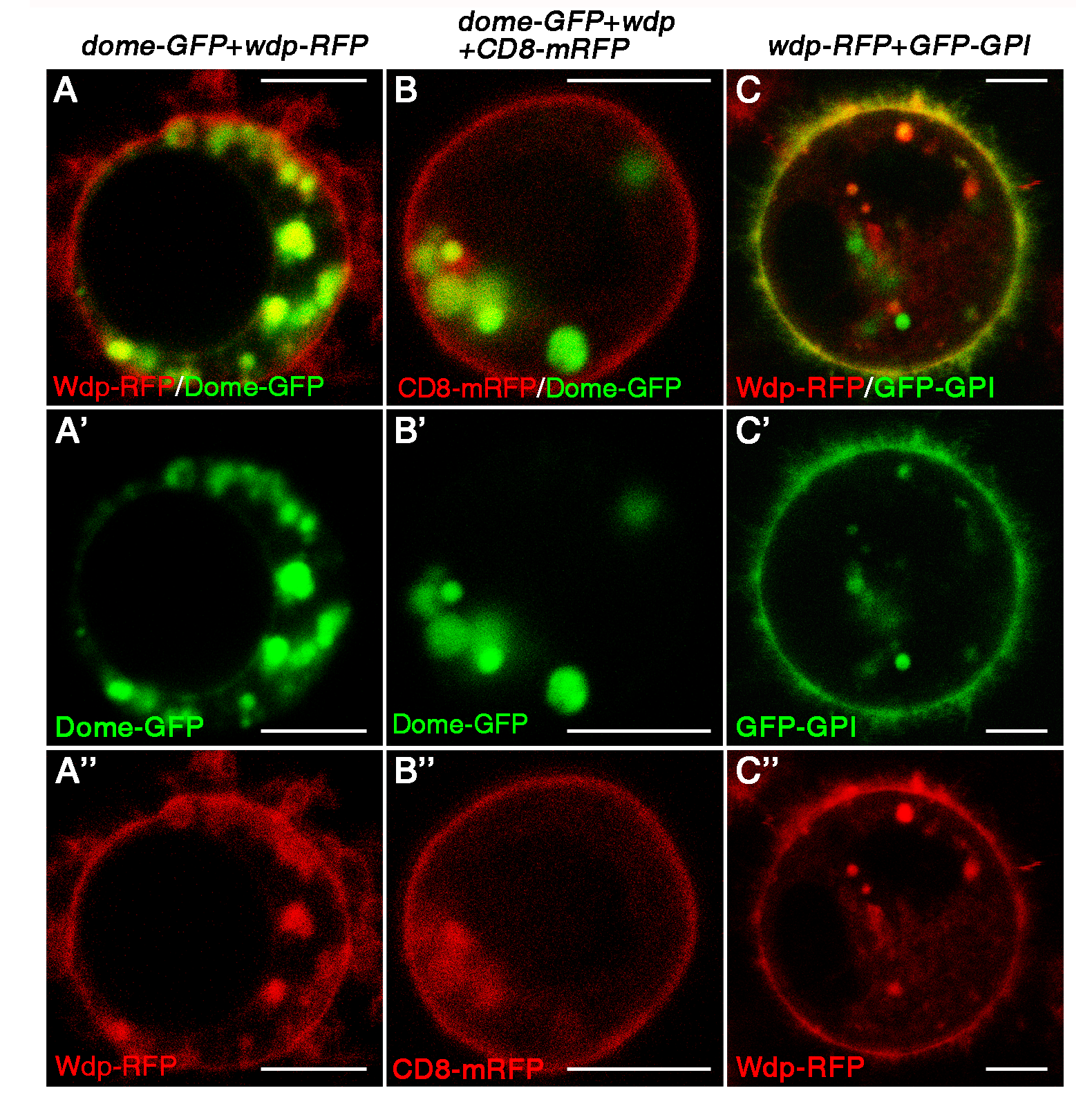

Supplement: S8 Fig — In live S2 cells cotransfected with dome-GFP and wdp-RFP vectors, the majority of Dome-GFP was localized as intracellular punctate structures (A and A’). However, the subcellular localization of other membrane proteins such as CD8-mRFP (B and B”) or GFP-GPI (C and C’) was not affected when coexpressed with Wdp, indicating that Wdp promotes Dome internalization without affecting the subcellular localization of other membrane molecules. Scale bars, 5μm. (TIF) [file pgen.1005180.s008.tif]

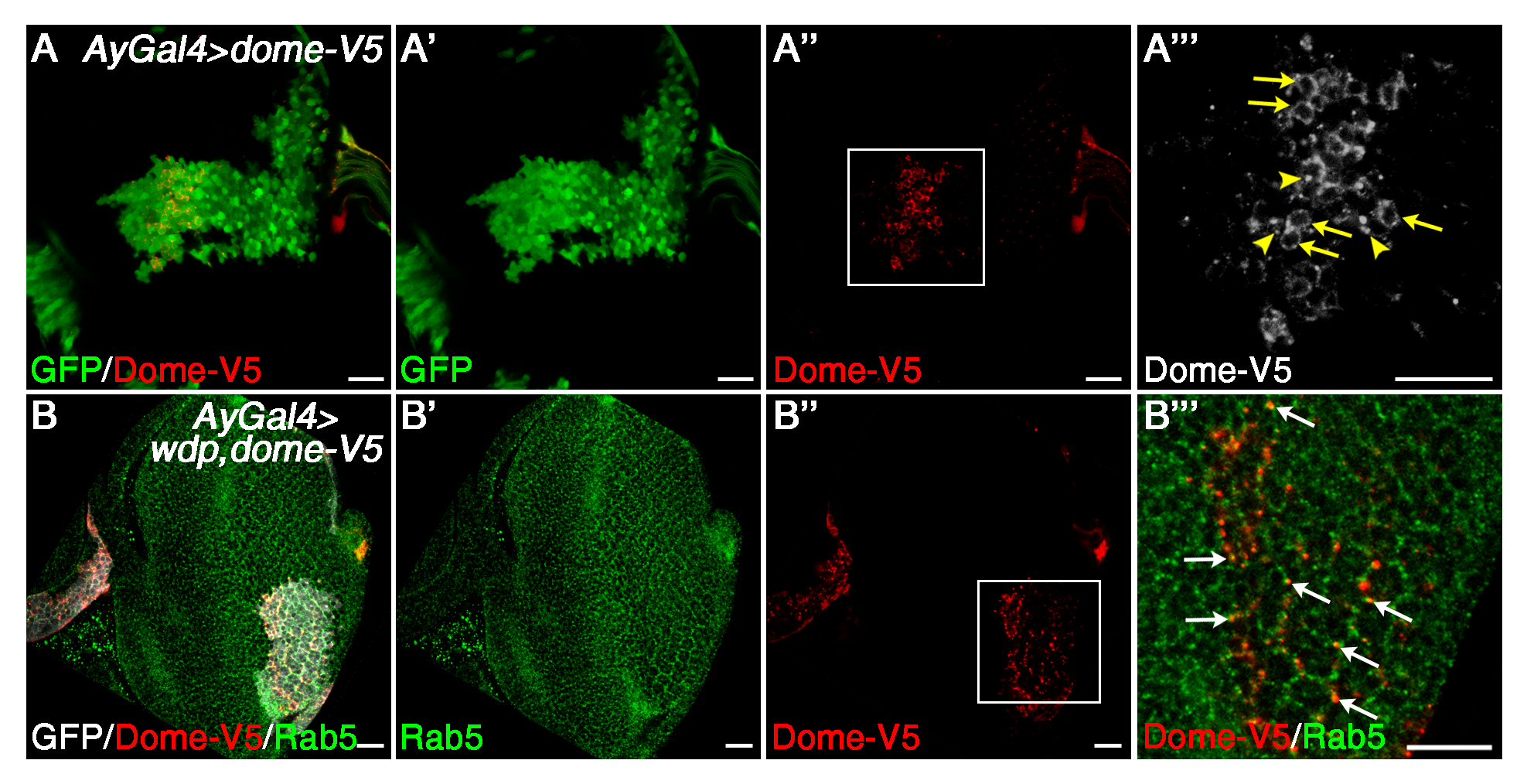

Supplement: S9 Fig — (A-A‴) In eye discs bearing GFP positively marked clones overexpressing Dome-V5 (Act>y+>Gal4, UAS-GFP, UAS-dome-V5), Dome-V5 was mainly localized on the cell membrane (yellow arrows) despite some intracellular punctate structures (yellow arrowheads). A‴ is the enlarged image of the position labeled by square box in A”. (B-B‴) In eye discs bearing GFP positively marked clones expressing Dome-V5 together with Wdp (Act>y+>Gal4, UAS-GFP, UAS-dome-V5, UAS-wdp), Dome-V5 was depleted from cell membrane but detected as cytoplasmic particles (B”), which were partially colocalized with early endosome marker Rab5 (B‴, white arrows). B‴ is the enlarged image of the position labeled by square box in B”. All the eye discs shown here are oriented posterior right. Scale bars, 20μm. (TIF) [file pgen.1005180.s009.tif]
