# Supplementary material for: Windpipe Controls Drosophila Intestinal Homeostasis by Regulating JAK/STAT Pathway via Promoting Receptor Endocytosis and Lysosomal Degradation
Source: PLoS Genet. 2015 Apr 29;11(4):e1005180. doi: 10.1371/journal.pgen.1005180 (PMC4414558; doi:10.1371/journal.pgen.1005180)
Supplement: S1 Table — In this ChIP assay, we totally got 1487 peaks with p-value<0.01. The above table shows partial putative JAK/STAT downstream targets. Some of them have previously been reported as potential targets or components of the JAK/STAT pathway through microarray or RNAi screening methods. The binding sites of STAT92E around the ChIP peaks (±500bp) include TTCNNGAA, TTCNNNGAA and TTCNNNNGAA. The full ChIP-Seq data can be found in the GEO database with the accession number GSE67346. (DOC) [file pgen.1005180.s010.doc]

**S1 Table**

Partial JAK/STAT targets identified from ChIP experiments with adult gut tissues

| **Target Genes** | **Peaks**  **(p<0.01)** | **Location of Peaks** | **STAT92E Binding Sites** | **Previous Reported**  **Evidence** |
| --- | --- | --- | --- | --- |
| *Socs36E* | 5 | chr2L:18150700-18150879 (1st intron)  18148850-18149199 (1st intron)  18144360-18144599 (1st intron)  18143400-18143619 (1st intron)  18140260-18140469 (3rd intron) | 7  4  7  3  2 |  |
| *dome* | 1 | chrX: 19577210-19577429 (5` UTR) | 2 |  |
| *Stat92E* | 2 | chr3R: 16376070-16376269 (1st intron)  16368450-16368579 (1st intron) | 2  0 |  |
| *chinmo* | 1 | chr2L: 1691120-1691289 (3rd intron) | 1 |  |
| *drs (drosomycin)* | 1 | chr3L: 3368290-3368529 (Upstream 1.1 Kb) | 2 |  |
| *pnt* | 4 | chr3R: 19163260-19163379 (3rd intron)  19162840-19163049 (3rd intron)  19142670-19142899 (3rd intron)  19122470-19122789 (6th intron) | 3  5  1  1 |  |
| *hyd* | 4 | chr3R: 5545310-5550519 (1st intron and  CDS)  5542180-5545119 (introns and CDS)  5541370-5542089 (introns and CDS)  5540660-5541229 (introns and CDS) | 11    6  0  0 |  |
| *laza* | 1 | chr3L: 22451700-22451989 (3` UTR) | 2 |  |
| *bsg* | 4 | chr2L: 8083270-8083369 (Upstream 50bp)  8088080-8088279 (1st intron)  8099760-8099919 (2nd intron)  8106160-8106279 (5th intron) | 3  1  1  1 |  |
| *th (DIAP1)* | 2 | chr3L: 16042730-16042829 (5` UTR)  16040360-16040579 (1st intron) | 3  1 |  |
| *Kay(DFos)* | 3 | chr3R: 25601530-25601699 (1st intron)  25602170-25602439 (1st intron)  25608970-25609289 (1st intron) | 1  3  2 |  |
| *zip* | 1 | chr2R: 20889520-20889749 (4th intron) | 1 |  |
| *siz* | 2 | chr3L: 21038790-21038959 (1st intron)  21067300-21067469 (CDS) | 0  1 |  |
| *nub(Pdm1)* | 1 | chr2L: 12590590-12590819 (1st intron) | 2 |  |
| *neb* | 1 | chr2L: 20078700-20078919 (1st intron) | 3 |  |
| *mld* | 2 | chr3R: 20430000-20430249 (3rd intron)  20442300-20442449 (CDS) | 3  1 |  |
| *dia* | 1 | chr2L: 20763440-20763599 (CDS) | 1 |  |
| *chic* | 2 | chr2L: 5980170-5980319 (1st intron)  5975460-5975739 (3rd intron) | 1  3 |  |
| ***wdp*** | **3** | **chr2R: 18200920-18201189 (Upstream 1.5 Kb)**  **18194720-18194899 (2nd intron)**  **18194370-18194529 (2nd intron)** | **3**  **4**  **4** |  |
| *MTA1-like* | 1 | chr3R: 1470500-1470749 (2nd intron) | 3 |  |
| *Smr* | 3 | chrX: 12635460-12635659 (1st intron)  12624020-12624159 (2nd intron)  12599730-12599879 (5th intron) | 2  5  0 |  |
| *Samuel* | 2 | chr2L: 11044080-11044219 (2nd intron)  11027300-11027459 (6th intron) | 1  1 |  |
| *PGRP-LA* | 2 | chr3L: 9317210-9317309 (Upstream 10Kb)  9305660-9305769 (Upstream 22Kb) | 1  3 |  |
| *par-1* | 1 | chr2R: 15369770-15369929 (14th intron) | 2 |  |
| *Not1* | 1 | chr2R:5453140-5453429 (downstream 0.2Kb) | 1 |  |
| *CG6051* | 2 | chr3R: 22958960-22959129 (1st intron)  22956910-22957049 (1st intron) | 5  0 |  |
| *CG5953* | 1 | chr2L: 16526100-16526339 (2nd intron) | 7 |  |
| *CG31343* | 2 | chr3R: 17571490-17571629 (1st intron)  17574740-17575059 (downstream 15 bp, and 0.6Kb upstream *CG31233*) | 0  2 |  |
| *CG5399* | 2 | chr3R: 11520600-11520789 (upstream 0.2Kb)  11524970-11525089 (downstream 2 Kb) | 1  2 |  |
| *CG10623* | 1 | chr2L: 18957150-18957429 (5’ UTR) | 3 |  |

**Table References:**

1. Buchon N, Broderick NA, Poidevin M, Pradervand S, Lemaitre B. Drosophila intestinal response to bacterial infection: activation of host defense and stem cell proliferation. Cell Host Microbe. 2009 Feb 19;5(2):200-11.

2. Bina S, Wright VM, Fisher KH, Milo M, Zeidler MP. Transcriptional targets of Drosophila JAK/STAT pathway signalling as effectors of haematopoietic tumour formation. EMBO Rep. 2010 Mar;11(3):201-7.

3. Baeg GH, Zhou R, Perrimon N. Genome-wide RNAi analysis of JAK/STAT signaling components in Drosophila. Genes Dev. 2005 Aug 15;19(16):1861-70.

4. Muller P, Kuttenkeuler D, Gesellchen V, Zeidler MP, Boutros M. Identification of JAK/STAT signalling components by genome-wide RNA interference. Nature. 2005 Aug 11;436(7052):871-5.

5. Flaherty MS, Zavadil J, Ekas LA, Bach EA. Genome-wide expression profiling in the Drosophila eye reveals unexpected repression of notch signaling by the JAK/STAT pathway. Dev Dyn. 2009 Sep;238(9):2235-53.

6. Vodovar N, Vinals M, Liehl P, Basset A, Degrouard J, Spellman P, et al. Drosophila host defense after oral infection by an entomopathogenic Pseudomonas species. Proc Natl Acad Sci U S A. 2005 Aug 9;102(32):11414-9.
