# Supplementary material for: Windpipe Controls Drosophila Intestinal Homeostasis by Regulating JAK/STAT Pathway via Promoting Receptor Endocytosis and Lysosomal Degradation
Source: PLoS Genet. 2015 Apr 29;11(4):e1005180. doi: 10.1371/journal.pgen.1005180 (PMC4414558; doi:10.1371/journal.pgen.1005180)
Supplement: S1 Text — Genotypes of flies used in Figs 1–8 and S1–S9 Figs are listed, followed by the information of primers used for RT-qPCR, constructing luciferase vectors and Rab5 dsRNA synthesis. In addition, the protocol of Rab5 dsRNA synthesis is also included. (DOC) [file pgen.1005180.s012.doc]

**Supplemental Experimental Procedures**

**Genotypes of Flies Used in Individual Figures**

Figure 1:

*esg Gal4, UAS-GFP, tubGal80^ts^*

*esg Gal4, UAS-GFP, tubGal80^ts^/UAS-upd*

*esg Gal4, UAS-GFP, tubGal80^ts^/UAS-upd, UAS-STAT*

*yw, hs-flp; Act> y+> Gal4-UAS-CD8-GFP/+; Dome RNAi/+*

*yw, hs-flp; Act> y+> Gal4-UAS-CD8-GFP/+; STAT RNAi/+*

Figure 2

*esg-lacZ*

*esg-lacZ-wdp^1^/wdp^1^*

*Dl-lacZ*

*wdp^1/1^; Dl-lacZ/+*

*Su(H)+GBE-lacZ*

*Su(H)+GBE-lacZ/+ ;wdp^1/1^*

*10×STAT GFP(Ⅲ)*

*wdp^1/1^ ; 10×STAT GFP(Ⅲ)/+*

Figure 3

*UAS-GFP*, *hsflp*; *FRT^42D^ tubGal80/ FRT^42D^*; *tubGal4/+*

*UAS-GFP, hsflp; FRT^42D^ tubGal80/ FRT^42D^-wdp^1^; tubGal4/+*

*UAS-GFP, hsflp; FRT^42D^ tubGal80/ FRT^42D^-wdp^1^; tubGal4/UAS-wdp*

*Su(H)+GBE-lacZ/+; esg Gal4, UAS-GFP, tubGal80^ts^*

*esg Gal4, UAS-GFP, tubGal80^ts^*

*Su(H)+GBE-lacZ/+; esg Gal4, UAS-GFP, tubGal80^ts^/+;wdp RNAi/+*

*esg Gal4, UAS-GFP, tubGal80^ts^/+;wdp RNAi/+*

*esg Gal4, UAS-GFP, tubGal80^ts^/UAS-upd*

*esg Gal4, UAS-GFP, tubGal80^ts^/UAS-upd;wdpRNAi/+*

Figure 4

*10×STAT GFP/+; mirror-Gal4>RFP/+*

*10×STAT GFP/+; mirror-Gal4>RFP/UAS-wdp*

*yw, hsflp; Act>y+>Gal4, UAS-mRFP/+;10×STAT GFP/+*

*yw, hsflp; Act>y+>Gal4, UAS-mRFP/+;10×STAT GFP/UAS-wdp*

*hsflp; FRT^G13^ tubGal80/ FRT^G13^;tubGal4,UAS-mRFP,10×STAT GFP/+*

*hsflp; FRT^G13^ tubGal80/ FRT^G13^-wdp^1^;* *tubGal4,UAS-mRFP,10×STAT GFP/+*

*Su(H)+GBE-lacZ/+; esg Gal4, UAS-GFP, tubGal80^ts^*

*Su(H)+GBE-lacZ/+; esg Gal4, UAS-GFP, tubGal80^ts^/+;UAS-wdp/+*

*Su(H)+GBE-lacZ/+; esg Gal4, UAS-GFP, tubGal80^ts^/+;STAT RNAi/+*

*Su(H)+GBE-lacZ/+; esg Gal4, UAS-GFP, tubGal80^ts^/+;Dome RNAi/+*

Figure 5

*10×STAT GFP/+; mirror-Gal4>RFP/UAS-hop*

*10×STAT GFP/UAS-wdp; mirror-Gal4>RFP/UAS-hop*

*Su(H)+GBE-lacZ/+; esg Gal4, UAS-GFP, tubGal80^ts^/+;UAS-hop/+*

*Su(H)+GBE-lacZ/+; esg Gal4, UAS-GFP, tubGal80^ts^/UAS-wdp；UAS-hop/+*

Figure 7

*yw, hs-flp; Act> y+> Gal4-UAS-CD8-GFP/+;dome-V5/+*

*yw, hs-flp; Act> y+> Gal4-UAS-CD8-GFP/UAS-wdp;dome-V5/+*

Figure S1

*esg-lacZ*

*Su(H)+GBE-lacZ*

*Dl-lacZ*

*w1118*

*wdp^1^*

*wdp^2^*

*EnGal4/+;wdp RNAi/+*

*UAS-GFP, hsflp; FRT^42D^ tubGal80/ FRT^42D^-wdp^1^; tubGal4/+*

Figure S2

*yw, hs-flp; Act> y+> Gal4-UAS-CD8-GFP*

*yw, hs-flp; Act> y+> Gal4-UAS-CD8-GFP/+; Dome RNAi/+*

*yw, hs-flp; Act> y+> Gal4-UAS-CD8-GFP/+; STAT RNAi/+*

*yw, hsflp122, UAS-GFP; TubGal4, FRT82B, TubGal80 /FRT82B*

*yw, hsflp122, UAS-GFP; TubGal4, FRT82B, TubGal80 / FRT82B-STAT92E06346*

*yw, hsflp, TubGal80, FRT19A/Notch^264-39^-FRT19A; Act-Gal4,UAS-GFP/+*

*yw, hsflp122, UAS-GFP/UAS-Notch RNAi; TubGal4, FRT82B, TubGal80 /FRT82B- STAT92E06346*

Figure S3

*w1118*

*wdp^1/1^*

*esg-lacZ/cyo*

*wdp^1^, esg-lacZ/wdp^2^*

Figure S4

*10×STAT GFP(Ⅲ)/+*

*wdp^1/1^; 10×STAT GFP(Ⅲ)/+*

*10×STAT GFP/+; mirror-Gal4>RFP/wdp RNAi*

*10×STAT DGFP*

*wdp^1/1^; 10×STAT DGFP*

*Myo1A Gal4;gal80^ts^-10×STAT GFP*

*Myo1A Gal4;gal80^ts^-10×STAT GFP/wdp RNAi*

Figure S5

*yw, hs-flp; Act> y+> Gal4-UAS-CD8-GFP/+;UAS-wdp/+*

Figure S6

*yw, hsflp; Act>y+>Gal4, UAS-mRFP/+;10×STAT GFP/+*

*yw, hsflp; Act>y+>Gal4, UAS-mRFP/UAS-upd;10×STAT GFP*

*yw, hsflp; Act>y+>Gal4, UAS-mRFP/UAS-upd;10×STAT GFP/UAS-wdp*

Figure S9

*yw, hs-flp; Act> y+> Gal4-UAS-CD8-GFP/+;dome-V5/+*

*yw, hs-flp; Act> y+> Gal4-UAS-CD8-GFP/UAS-wdp;dome-V5/+*

**Primers used for RT-qPCR**

wdp-S: TGGCAACCACAATGAGGAACAG

wdp-AS: GACCGAGAAGACCTTCCAGTCAAC

Gp150-S: CTAATCCACGCCAGCCACTC

Gp150-AS: TGGTCGTCTCCACGGGTAAATC

Rab5-S: CTGCTGTTCATGGAAACCTC

Rab5-AS: CTTAGGTAGTTTCTTGGCAATGG

RpL11-S: GGTCCGTTCGTTCGGTATTCGC

RpL11-AS: GGATCGTACTTGATGCCCAGATCG

**Primers used for constructing luciferase vectors in Figure 1C:**

The following primers were used and the transcription start point of *wdp* was labeled as 0.

Luc-*wdp*-BS1 F (-2371): AGGCACGAAGTTTCACCCTACTGTAC,

Luc-*wdp*-BS1 R (-591): ACACACTCTCTTCTTGGGCAACAGC;

Luc-*wdp*-BS2 F (4253): GAAAAAACACAATCAACTTATCATAAATCTAG,

Luc-*wdp*-BS2 R (5701): TTTCGTACTTGGCTAGTTATTTGACC;

Luc-*wdp*-BS3 F (5845): GACTATTAGTATGCACAAACAGCAGGAT,

Luc-*wdp*-BS3 R (7306): CTTTTAGCTTGGGTTCACTTCGC;

Luc-*wdp*-BS4 F (9919): GAGGCTCAGGGCTTATCAATGC,

Luc-*wdp*-BS4 R (11191): CGGCACCGTTGACTGGAAGG

**Primers used for Rab5 dsRNA synthesis:**

Rab5 dsRNA-1 F: taatacgactcactatagggGCTCGTTTCGTTTCGTTTCT

Rab5 dsRNA-1 R: taatacgactcactatagggCTATCTTAGGCCTTGCGACC

Rab5 dsRNA-2 F: taatacgactcactatagggACGATAGGTGCGGCCTTTCTGAC

Rab5 dsRNA-2 R: taatacgactcactatagggCGTCTTGGCGGAGGTTTCCATG

lacZ dsRNA F: taatacgactcactatagggGCATAAACCGACTACACAAATCAG

lacZ dsRNA R: taatacgactcactatagggGGCTTCATCCACCACATACAG

**Rab5 dsRNA synthesis**

dsRNAs were synthesized according to standard protocols. Both sense and antisense RNAs were synthesized from a single PCR product using the T7 MegaScript RNA polymerase (Ambion). To effectively knock down target genes, S2 cells were treated with dsRNAs directed against lacZ (control) or Rab5 (Rab5 dsRNA-1 or Rab5 dsRNA-2) for 5 days.
